# Behavioral Economic Framing for Enrollment and Retention of Patients in Remote Blood Pressure Monitoring: A Randomized Clinical Trial

**DOI:** 10.1001/jamanetworkopen.2025.29825

**Published:** 2025-09-02

**Authors:** Shivan J. Mehta, Joseph Teel, Evelyn Okorie, Catherine Reitz, Alison Purcell, Christopher K. Snider, Kayla Clark, Rebecca C. Kersting, Karen Glanz, Mary Putt, Charles Rareshide, Kevin G. Volpp

**Affiliations:** 1 Department of Medicine, Perelman School of Medicine, University of Pennsylvania, Philadelphia; 2 Center for Health Care Transformation and Innovation, University of Pennsylvania, Philadelphia; 3 Center for Health Incentives and Behavioral Economics, Leonard Davis Institute of Health Economics, University of Pennsylvania, Philadelphia; 4 Department of Family Medicine and Community Health, Perelman School of Medicine, University of Pennsylvania, Philadelphia; 5 Department of Biostatistics, Epidemiology, and Informatics, Perelman School of Medicine, University of Pennsylvania, Philadelphia; 6 Department of Health Care Management, the Wharton School, University of Pennsylvania, Philadelphia

## Abstract

**Importance:**

Remote blood pressure (BP) monitoring for hypertension has been limited by low participation and engagement.

**Objective:**

To evaluate if an opt-out behavioral economic approach to remote BP monitoring improves enrollment and BP outcomes compared with an opt-in approach.

**Design, Setting, and Participants:**

This pragmatic, 3-arm randomized clinical trial included patients aged 18 to 75 years with hypertension who were followed up by an academic family medicine practice in Philadelphia. Eligible patients used text messaging, had at least 2 elevated BP measurements during the prior 24 months, and were prescribed hypertension medication. Patients were randomized beginning February 25, 2021, and the last patient completed the program March 22, 2022. Data were analyzed from December 2023 to January 2024.

**Interventions:**

Prior to consent, patients were randomized 2:2:1 to opt-in or opt-out recruitment for remote monitoring of BP or to usual-care control. The opt-in group received outreach to consent and enroll in remote monitoring, and those who consented received a home BP monitor; the opt-out group received a home BP monitor and similar recruitment and follow-up. For 6 months, participants in the opt-in and opt-out groups received weekly text messages requesting BP readings and received support from a social partner or clinician as needed.

**Main Outcomes and Measures:**

The primary outcome was the proportion of all participants in the intervention arms consenting to enroll in remote BP monitoring. Secondary outcomes for the intervention groups included number of BP measurements submitted and proportion of patients actively engaged. Secondary outcomes for all participants were BP measurements and the proportion with controlled BP.

**Results:**

Among 424 randomized patients (171 opt-in, 168 opt-out, and 85 control), the mean (SD) age was 52.1 (11.5) years, and 264 (62.3%) were female. A total of 58 patients (33.9%) in the opt-in and 63 (37.5%) in the opt-out arm consented to enroll in monitoring, yielding no significant difference in enrollment rate (3.6 percentage points [pp]; 90% CI, −5.0 to 12.1 pp; *P* = .49). There was no difference in the mean number of BP measurements submitted (unadjusted difference, −0.03 [95% CI, −0.09 to 0.03] measurements; *P* = .30) or proportion of actively engaged patients (absolute difference, −0.7 pp [90% CI, −15.6 to 14.3 pp]; *P* = .94) between intervention arms. Using BP measurements from clinic visits, 55 patients (32.2%) in the opt-in arm and 64 (38.1%) in the opt-out arm had controlled BP, compared with 18 (21.2%) in the control arm (opt-in difference vs control, 11.7 pp [95% CI, −0.2 to 23.5 pp]; *P* = .05; opt-out difference vs control, 18.0 pp [95% CI, 6.1-30.0 pp]; *P* = .003).

**Conclusions and Relevance:**

In this randomized clinical trial, the behavioral economic approach of opt-out framing vs opt-in framing did not improve enrollment and retention of patients in a remote BP monitoring program. The findings suggest additional approaches are needed to boost participation.

**Trial Registration:**

ClinicalTrials.gov Identifier: NCT04714398

## Key Points

**Question:**

Can sending a blood pressure (BP) monitor to patients with hypertension and using opt-out rather than opt-in framing improve enrollment and engagement in a remote BP monitoring program?

**Findings:**

In this randomized clinical trial of 424 patients with hypertension, both opt-out and opt-in framing yielded similar rates of enrollment in a remote BP monitoring program.

**Meaning:**

The findings suggest use of opt-out behavioral economic framing may not improve patient participation in remote BP monitoring programs.

## Teaser

This randomized clinical trial investigates whether opt-out compared with opt-in behavioral economic framing improves enrollment and engagement in remote blood pressure monitoring for adults with hypertension.

## Introduction

Hypertension is a leading cause of morbidity and mortality from cardiovascular disease.^1,2,3^ Despite effective therapies to treat hypertension, only about half of patients with hypertension have good blood pressure (BP) control.^4^ Remote monitoring of BP has shown effectiveness in many clinical trials, but widespread implementation has been limited by enrollment and integration into clinical practice.^5,6,7,8,9,10,11,12,13^ For remote monitoring to be effective, patients need to participate, and the information needs to be acted on in a way that is timely but does not overburden primary care clinicians.

There is an opportunity to increase participation in remote BP monitoring by incorporating insights from the field of behavioral economics, which acknowledges that humans have systematic biases in thinking that can be harnessed to improve healthy behavior.^14,15,16^ Shifting from opt-in to opt-out framing has dramatically increased participation in cancer screening and vaccinations.^17,18,19^ The endowment effect indicates that people place more value on something they possess as opposed to something they could obtain.^20^ In addition, humans are influenced by others through social support, which can include encouragement from friends or family members.^21,22^ In the case of remote BP monitoring, sending a BP cuff with an invitation and including opt-out framing and social support could increase enrollment, engagement, and effectiveness of the program. In this trial, we evaluated whether opt-out framing after providing remote BP monitoring cuffs to patients with hypertension preenrollment increased both enrollment in the program and clinical effectiveness of a remote BP monitoring intervention.

## Methods

### Study Design

This was a 3-arm randomized clinical trial applying behavioral approaches to encourage patients with hypertension to manage their BP using a remote monitoring program. Eligible patients were randomized in a 2:2:1 ratio into 3 arms: (1) opt-in recruitment, (2) opt-out recruitment (including a BP cuff provided preenrollment), or (3) control. The study was approved by the institutional review board (IRB) at the University of Pennsylvania. A waiver of informed consent for the enrollment phase of the study was obtained because this phase of the study posed minimal risk to patients and could not have been carried out practicably without the waiver.^23^ Participation in remote monitoring required patients to provide oral consent via telephone. The IRB waived informed consent for patients in the control arm because there was low risk and obtaining consent would have precluded evaluation of usual care. The study followed the Consolidated Standards of Reporting Trials (CONSORT) reporting guideline. The protocol and statistical plan appear in Supplement 1.

### Study Population

The study included patients followed up by a large family medicine practice at an urban academic health system in Philadelphia. We included patients between the ages of 18 and 75 years who had text messaging capability, a diagnosis of hypertension, at least 2 visits to the practice within the past 2 years, and at least 2 BP measurements that exceeded Eighth Joint National Committee hypertension guidelines (including at the most recent visit at the time of medical record review) and who were prescribed at least 1 medication for hypertension.^24^ We excluded patients with a body mass index (BMI, calculated as weight in kilograms divided by height in meters squared) greater than or equal to 50, since they would likely require a different BP cuff; patients with a history of metastatic (stage 4) cancer, end-stage kidney disease, heart failure, and/or dementia, as their participation in the trial may not be clinically appropriate; and patients designated as non–English speaking and requiring a translator, as the intervention was text-based.

Race and ethnicity data, included in the analysis because there are known disparities in engagement and outcomes for hypertension,^1,4^ were based on self-reported data in the EHR. Race categories were Asian, Black, White, and other (included Pacific Islander, multiracial, or other race) or unknown (included patients with missing data or who self-identified their race as “unknown”). Ethnicity categories were Hispanic or Latino and not Hispanic or Latino.

### Recruitment and Randomization

Patients were identified through automated data extraction from the electronic health record (EHR) in February 2021, with additional medical record review performed by research staff to confirm eligibility. In variable blocks of 5 and 10 using a computer-generated algorithm, eligible patients were randomized in a 2:2:1 ratio to opt-in, opt-out, and control.

Patients in the opt-in recruitment arm received an invitation letter and up to 3 recruitment telephone calls to obtain verbal consent for remote monitoring. After consent, the research coordinators (E.O., K.C.) mailed a BP cuff and initiated the remote monitoring program. Patients were also asked (but not required) to provide names of up to 3 friends or family members to serve as a support partner, who then provided verbal agreement to participate but not formal consent.

Patients assigned to the opt-out recruitment arm received an electronic BP cuff (OMRON 3 Series [OMRON Healthcare Inc], or Welch Allyn 1700 Series [Welch Allyn Inc] if BMI >40) with a letter describing the program and opt-out framing regarding their participation. Patients in this arm received similar recruitment and follow-up to the opt-in arm.

Patients in the control arm were not contacted by study staff and received usual care, which involved management of hypertension by the PCP, typically through routine office visits. Eligible patients were randomized beginning February 25, 2021, and enrolled in remote monitoring between March 4, 2021, and September 22, 2021. Patients were enrolled on a rolling basis, and the last enrolled patient completed the program March 22, 2022. Investigators were blinded to arm allocation, but research staff were not, as they needed to administer the intervention. The trial was pragmatic in its integration into clinical operations and the EHR, and BP outcomes measured from routine visits.

### Interventions

For 6 months, all patients in the remote monitoring groups received text message prompts 3 times per week asking to input BP and once per week asking how often they had taken their BP medication. They also received facts about BP and lifestyle changes and feedback regarding their BP control.

Escalations occurred in the following scenarios: (1) chronic escalations (3 out of 10 BP readings above guidelines),^25^ (2) severely high escalations (any single systolic BP [SBP] measurement ≥180 mm Hg and/or diastolic BP [DBP] measurement ≥110 mm Hg), or (3) severely low escalations (any single SBP measurement <90 mm Hg and/or DBP measurement <50 mm Hg). Escalations were noted to the patient via text message and routed to either the nursing pool (chronic escalations) or directly to the nurse practitioner (A.P.) (severely high or low escalations). All patient-submitted BP measurements were visible in the EHR BP flow sheet.

A support partner was added to the program after a patient’s first chronic escalation to help provide encouragement. The partner received weekly updates about the patient’s medication adherence and BP monitoring and was notified if the patient’s BPs were routinely elevated above a certain threshold. The partner was encouraged to talk to the patient about BP control and had the opportunity to opt out of a weekly support message sent to the patient on the partner’s behalf.

If a patient’s BP measurements triggered a second escalation, a nurse or the nurse practitioner could contact them via the study text-messaging platform to obtain additional information, provide additional guidelines for BP management, or set up a telephone consultation. If BP measurements triggered a third escalation, the nurse practitioner was encouraged to consider changing the dosing of the patient’s existing medications, change their medications, or prescribe additional medications to help manage their BP. Patients nonresponsive to texting prompts were contacted by the study team with reengagement attempts after 7, 14, 21, and 35 consecutive missed prompts. All text-messaging content appears in the protocol in Supplement 1.

### Study Outcomes

The primary outcome was the proportion of patients consenting to receive remote monitoring out of the total number randomized. For the intervention arms, secondary outcomes included the proportion of the requested BP measurements submitted by each patient and the proportion of patients actively engaged with the program (defined as submitting at least 50% of the requested BP measurements), based on a prior trial.^26^ For participants in all arms, we compared the mean number of BP measurements recorded in the EHR between 6 and 12 months after randomization and the final SBP level in the control vs intervention arms.

### Statistical Analysis

An intention-to-treat analysis was used for the primary outcome and the secondary outcomes of controlled BP and actual BP measurements collected from the EHR. For the intervention arms, secondary outcomes (engagement with the program, BP control) were based on participants who consented postrandomization. The primary analysis compared the proportion of patients participating in each study arm using a *Z* test with 90% CIs, as this was a pilot study (type I error rate was .10). The study will inform enrollment rates that can be used to guide study design and enrollment goals for future trials. For a sample size of 170 per opt-in and opt-out group, if the observed difference in rates was 10% and the rate for the opt-in group was 30%, the 90% CI would have a half-width of 9.0%, with boundaries of 1.0% and 19.0%. Additionally, using a 2-sided type I error rate of .10, this study design had 80% power to detect an absolute difference of 12.9 percentage points (pp) between the opt-out and opt-in arms. Except for the primary outcome, we used 95% CIs as per convention.

For the primary end point, we also conducted a logistic regression model with adjustment for demographic and clinical characteristics (age, BMI, sex, race, insurance type, and diabetes and kidney disease status). We also compared the mean rates of participation in BP measurements using a logistic regression model adjusted for the number of BP measurements requested as well as demographic and clinical variables. Similarly, we estimated the odds of engagement (defined as submitting at least 50% of the requested BP measurements over the duration of the program), with adjustment for baseline variables.

We estimated the total proportion of patients with controlled BP in the intervention and control arms, using all randomized participants as the denominator to follow intention-to-treat principles. Using a linear model, we determined whether mean BP differed by arm after adjustment for baseline BP and used the model to provide estimates of both final BP and changes in BP from baseline. To address missing data, we conducted multiple imputation of the BP data using a monotone predicted mean matching method in SAS Studio, version 3.82 (SAS Institute Inc), fit to each of the 20 imputations and combined using Rubin method. The variables for imputation included study arm, age, BMI, sex, race, insurance status, diabetes, chronic kidney disease, and eligibility BP. Data were analyzed from December 2023 to January 2024 using SAS Studio, version 3.82. Two-sided *P* < .05 was considered significant.

## Results

### Patient Characteristics

A total of 620 patients in the medical record reviews had confirmed eligibility, and 425 were randomized (171 to opt-in, 168 to opt-out, and 85 to control); 1 patient was erroneously randomized and removed from the study before outreach (Figure). The mean (SD) patient age was 52.1 (11.5) years (range, 23-75 years); 264 (62.3%) were female and 160 (37.7%) were male. A total of 135 (31.8%) were insured by Medicaid and 203 (47.9%) had private insurance or were self-paying. Eleven patients (2.6%) were Asian, 365 (86.1%) were Black, 33 (7.8%) were White, and 15 (3.5%) were of other or unknown race; 13 (3.1%) identified as Hispanic or Latino and 407 (96.0%) as not Hispanic or Latino, and 4 (0.9%) had unknown ethnicity (Table 1). A total of 139 of the 424 patients randomized (32.7%) did not have a clinic visit during the study and were therefore missing data on BP.

**Figure.**
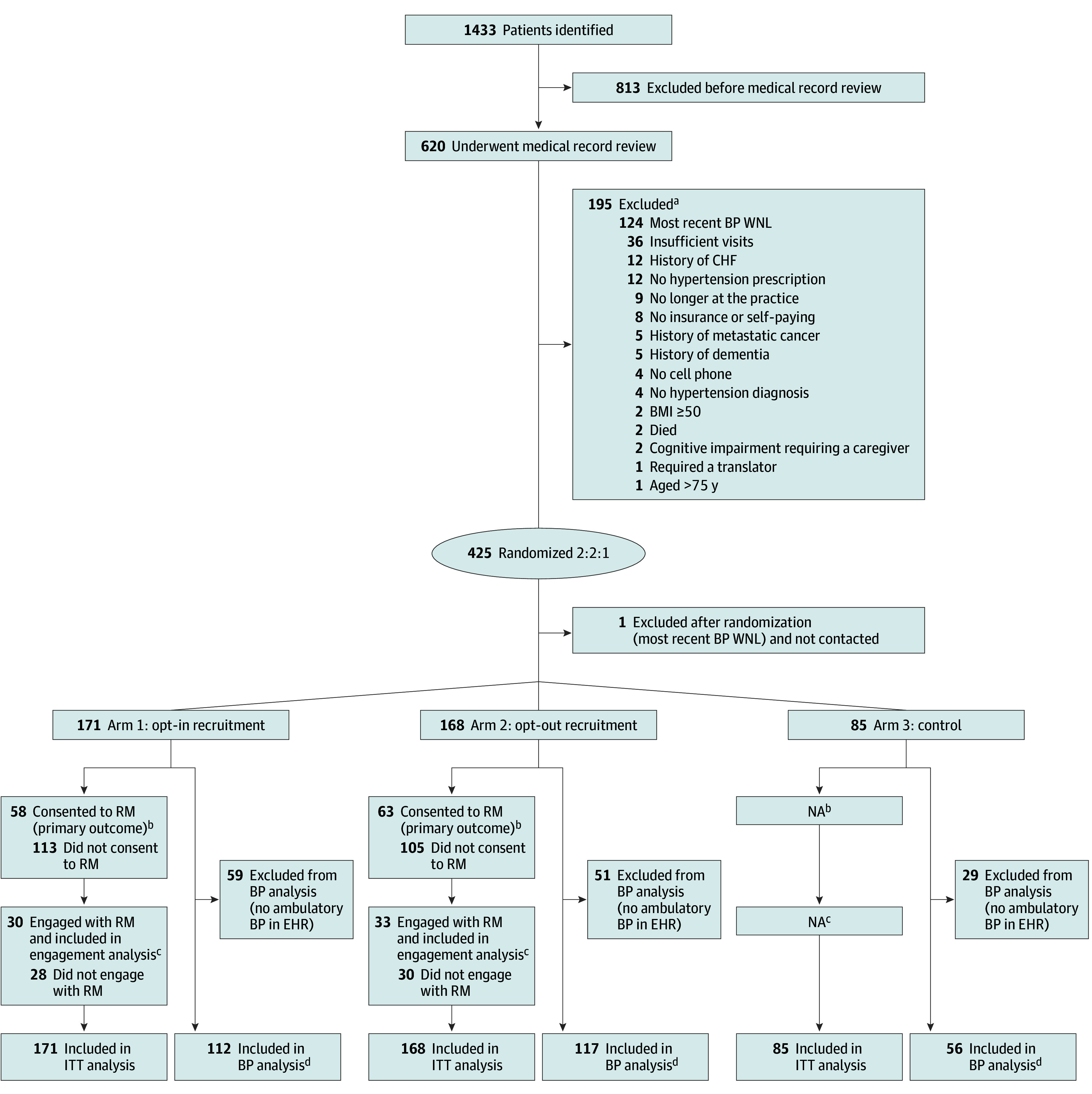
Consolidated Standards of Reporting Trials Flow Diagram BMI indicates body mass index (calculated as weight in kilograms divided by height in meters squared); BP, blood pressure; CHF, congestive heart failure; EHR, electronic health record; ITT, intention to treat; NA, not applicable; WNL, within normal limits. ^a^Some patients were excluded for more than 1 reason. ^b^The primary outcome was the number of patients consenting to remote monitoring (RM) out of the number randomized and was not applicable to patients in the control arm. ^c^Engagement analyses included the number of patients actively engaged with the RM program (submitting at least 50% of requested BP measurements) out of the total number consenting to RM and were not applicable to patients in the control arm. ^d^Patients included in the BP analysis had at least 1 ambulatory BP measurement in the EHR between 6 and 12 months from the date of randomization.

**Table 1.** Participant Characteristics

Characteristic	Participants^a^			
	Study arm			Overall (N = 424)
	Opt-in (n = 171)	Opt-out (n = 168)	Control (n = 85)	
Age, mean (SD), y	52.2 (12.1)	52.4 (11.5)	51.5 (10.2)	52.1 (11.5)
Sex				
Female	108 (63.2)	102 (60.7)	54 (63.5)	264 (62.3)
Male	63 (36.8)	66 (39.3)	31 (36.5)	160 (37.7)
Race				
Asian	7 (4.1)	1 (0.6)	3 (3.5)	11 (2.6)
Black	143 (83.6)	146 (86.9)	76 (89.4)	365 (86.1)
White	16 (9.4)	13 (7.7)	4 (4.7)	33 (7.8)
Other or unknown^b^	5 (2.9)	8 (4.8)	2 (2.6)	15 (3.5)
Ethnicity				
Hispanic or Latino	7 (4.1)	2 (1.2)	4 (4.7)	13 (3.1)
Not Hispanic or Latino	163 (95.3)	163 (97.0)	81 (95.3)	407 (96.0)
Unknown	1 (0.6)	3 (1.8)	0	4 (0.9)
Insurance				
Private or self-pay	94 (55.0)	70 (41.7)	39 (45.9)	203 (47.9)
Medicare	36 (21.1)	39 (23.2)	11 (12.9)	86 (20.3)
Medicaid	41 (24.0)	59 (35.1)	35 (41.2)	135 (31.8)
BMI, mean (SD)	33.5 (6.1)	32.9 (6.4)	33.8 (6.6)	33.3 (6.3)
Diabetes	68 (39.8)	66 (39.3)	28 (32.9)	162 (38.2)
Kidney disease	14 (8.2)	23 (13.7)	8 (9.4)	45 (10.6)
Eligibility systolic BP, mean (SD)	151.2 (14.1)	152.3 (14.1)	149.8 (12.2)	151.4 (13.7)
Eligibility diastolic BP, mean (SD)	89.3 (11.7)	88.0 (14)	90.1 (11.2)	89.0 (12.6)

Abbreviations: BMI, body mass index (calculated as weight in kilograms divided by height in meters squared); BP, blood pressure.

^a^ Data are presented as number (percentage) of participants unless otherwise indicated.

^b^ Includes patients who self-identified as Pacific Islander, other race, or multiracial in the electronic health record. Unknown includes those missing data and those who self-identified as unknown.

### Recruitment Outcomes

Among patients randomized, 58 (33.9%) in the opt-in arm and 63 (37.5%) in the opt-out arm consented to participate (Figure). The participant characteristics of those who consented within each intervention arm were generally similar, with the notable exception of insurance (eTable 1 in Supplement 2). We found no statistically significant difference in the rate of consent between arms (3.6 pp; 90% CI, −5.0 to 12.1 pp; *P* = .49) (Table 2). Neither the unadjusted odds of consent (odds ratio [OR], 1.17; 95% CI, 0.75-1.82; *P* = .49) nor the odds of consent adjusted for age, BMI, sex, race, insurance type, diabetes, and kidney disease status (adjusted OR [AOR], 1.16; 95% CI, 0.73-1.84; *P* = .54) were significantly different from the control group. Out of the 121 patients that enrolled, 58 (47.9%) identified a support partner, and 9 of those identified (15.5%) enrolled as a partner.

**Table 2.** Rates and Odds of Consent for Intervention Arms

	Participants, No. (%)		Effect			
	Opt-in (n = 171)	Opt-out (n = 168)	Rate difference (90% CI), pp^a^	*P* value	OR (90% CI)	*P* value
Consented (unadjusted)	58 (33.9)	63 (37.5)	3.6 (−5.0 to 12.1)	.49	1.17 (0.75-1.82)	.49
Consented (adjusted)^b^	NA	NA	NA	NA	1.16 (0.73-1.84)	.54

Abbreviations: NA, not applicable; OR, odds ratio; pp, percentage points.

^a^ Difference in opt-out vs opt-in rates.

^b^ Adjusted for age, body mass index, sex, race, insurance type, and diabetes and kidney disease status.

### Engagement Outcomes

The opt-in arm submitted a mean (SD) of 40.5 (28.1) BP measurements and the opt-out arm submitted 39.3 (27.2). The mean number of BP measurements submitted was not significantly different between arms in unadjusted (difference, −0.03 [95% CI, −0.09 to 0.03] measurements; *P* = .30) or adjusted (difference, −0.04 [95% CI, −0.10 to 0.02] measurements; *P* = .16) analyses (eTable 2 in Supplement 2). The proportion of actively engaged patients (submitting at least 50% of requested BP measurements) was nearly identical between intervention arms: 30 of the 58 patients (51.7%) in the opt-in arm were considered actively engaged vs 33 of 63 (52.4%) in the opt-out arm, an absolute difference of −0.7 pp (90% CI, −15.6 to 14.3 pp; *P* = .94) (eTable 3 in Supplement 2).

### BP Outcomes

There were similar proportions of patients with clinic BP readings in the opt-in (112 [65.5%]), opt-out (117 [69.6%]), and control (56 [65.9%]) arms. However, using intention-to-treat analyses, 55 patients (32.2%) in the opt-in and 64 (38.1%) in the opt-out study arms showed BP control, compared with 18 (21.2%) in the control arm. Compared with the control arm, patients in the opt-out but not the opt-in arm had significantly higher rates of showing BP control (opt-in difference, 11.7 pp [95% CI, −0.2 to 23.5 pp]; *P* = .05; opt-out difference, 18.0 pp [95% CI, 6.1-30.0 pp]; *P* = .003) (Table 3). An analysis using multiple imputation to address missing data yielded similar findings, as did a random-effects model to account for individuals with multiple BP measurements.

**Table 3.** Differences in Rates of Controlled Blood Pressure by Study Arm^a^

Model	Opt-in arm		Opt-out arm	
	Difference (95% CI), pp	*P* value	Difference (95% CI), pp	*P* value
Control arm as reference				
Unadjusted	11.7 (−0.2 to 23.5)	.05	18.0 (6.1 to 30.0)	.003
Adjusted^b^	12.9 (0.1 to 24.8)	.03	19.9 (7.9 to 31.9)	.001
Opt-in arm as reference				
Unadjusted	NA	NA	6.3 (−3.3 to 16.0)	.20
Adjusted^b^	NA	NA	7.0 (−2.8-16.8)	.16

Abbreviation: NA, not applicable.

^a^ Controlled blood pressure (BP) was defined as systolic BP less than 140 mm Hg and diastolic BP less than 90 mm Hg.

^b^ Adjusted for age, body mass index, sex, race, insurance type, and diabetes and kidney disease status.

The mean (SD) eligibility SBP was 151.2 mm Hg (14.1 mm Hg) in the opt-in, 152.3 mm Hg (14.1 mm Hg) in the opt-out, and 149.8 mm Hg (12.2 mm Hg) in the control arm. Compared with the final SBP level in the control arm (mean [SD], 143.9 [15.2] mm Hg), final SBP levels were lower in the opt-in (mean [SD], 139.4 [13.5] mm Hg; difference vs control: −4.77 mm Hg [95% CI, −9.19 to −0.35 mm Hg]; *P* = .03) and opt-out (mean [SD], 138.5 [14.7] mm Hg; difference vs control: −6.26 mm Hg [95% CI, −10.67 to −1.84 mm Hg]; *P* = .006) arms (Table 4). Similar results were found when adjusting for demographic and clinical characteristics and in analyses with random effects or multiple imputation. With the same adjustments, final DBP levels in the opt-in (difference, −1.77 mm Hg; 95% CI, −4.60 to 1.06 mm Hg; *P* = .22) and opt-out (difference, −2.09 mm Hg; 95% CI, −4.92 to 0.74 mm Hg; *P* = .15) arms were not significantly different from the control group (eTable 4 in Supplement 2). Patient-submitted BP readings are shown in eTable 5 in Supplement 2, and patient reported medication adherence is shown in eTable 6 in Supplement 2.

**Table 4.** Differences in Mean Systolic Blood Pressure by Study Arm

Model	Opt-in arm		Opt-out arm	
	Difference, mean (95% CI), mm Hg	*P* value	Difference, mean (95% CI), mm Hg	*P* value
Control arm as reference				
Unadjusted	−4.77 (−9.19 to −0.35)	.03	−6.26 (−10.67 to −1.84)	.006
Adjusted^a^	−4.64 (−9.12 to −0.16)	.04	−6.51 (−10.99 to −2.02)	.005
Imputed^b^	−4.7 (−9.3 to 0.0)	.048	−5.9 (−10.4 to −1.3)	.01
Opt-in arm as reference				
Unadjusted	NA	NA	−1.48 (−5.07 to 2.11)	.42
Adjusted^a^	NA	NA	−1.87 (−5.58 to 1.84)	.32
Imputed^b^	NA	NA	−1.2 (−4.6 to 2.2)	.49

Abbreviation: NA, not applicable.

^a^ Adjusted for age, body mass index, sex, race, insurance type, and diabetes and kidney disease status.

^b^ Imputed with 20 imputations using a monotone predicted mean matching method.

## Discussion

In this pragmatic randomized clinical trial, participation among patients receiving the opt-out framing, by mailing the BP monitor before enrollment, was not substantially increased compared with those receiving opt-in framing. However, both intervention groups, who were offered remote BP monitoring, showed improved BP control compared with the control group. Of note, the intention-to-treat population included both those who enrolled in the BP monitoring and those who did not, enabling evaluation of the effect of the intervention when offered in routine clinical practice.

Prior studies have shown that sending screening kits, a laboratory order, and electronic pill bottles with outreach can increase participation by framing participation as opt-out and leveraging reciprocity.^18,27,28,29,30^ There are several possible reasons why this trial did not show improved enrollment among the group that received the BP monitor ahead of time. First, this was not a true opt-out program, as the patients still needed to answer the telephone and consent to monitoring before they were enrolled. Second, the messaging about consent was similar between arms, so patients may not have thought the intervention was part of routine care (as it would be when implemented in clinical practice). Additionally, the prior outreach studies did not include a telephone call or consent component. While our study was designed to detect fairly large effects, as it had 80% power to detect about a 13-pp increase in enrollment, the estimated difference between arms of 3.6 pp (90% CI, −5.0 to 12.1 pp) is consistent with a null improvement from the opt-out intervention. In addition, limited enrollment rates might reflect our strategy of reaching out directly to patients not undergoing BP control, without referral from their primary care practitioner (PCP).

There has been mixed evidence of effectiveness in studies of remote monitoring for hypertension, with heterogeneity in the design of interventions and integration into clinical practice.^5,6,7,8,10,31^ In our own health system, 2 trials of text message engagement were conducted that required research consent to participate but did not show improved BP control. The first trial provided text messaging reminders for medication adherence, but there was no BP monitoring or connection to a clinician.^32^ The second trial provided remote BP monitoring, but results outside the healthy range were first sent directly to the PCP, who lacked capacity to manage the alerts outside of office visits; halfway through the study, based on feedback from the clinicians, the results were sent to a dedicated nurse practitioner.^26^ The improvement seen in BP control among patients in the current study could be attributed to several factors. First, the dedicated escalation pathway approached care in a stepped manner, so that initially, a nurse talked to the patients about adherence, and then a dedicated nurse practitioner could prescribe medications. Second, this study included additional behavioral interventions, such as social support and feedback. Third, this study did not require a separate BP visit for the final outcome measure. In prior studies, the control group was consented and required to participate in an end-of-study visit for BP measurement, which in itself may have acted as an intervention.^25,26^ This study’s findings also suggest that even those patients who were contacted but not consented to participate in remote monitoring had slightly better BP control than patients in the control arm, as the outreach (and the BP monitor in the opt-out arm) may have provided a nudge for patients to take their medications and control their BP. Fourth, there was a longer follow-up time frame of 6 months, compared with 4 months in prior studies.^26,32^

### Strengths and Limitations

The main strength of this study was its pragmatic design that was integrated into routine practice, with a technology platform embedded in the EHR and outcomes that were obtained from routine office visits. Therefore, the results likely simulate what would happen if the intervention were implemented in clinical practice. Intention-to-treat analyses were possible for the primary outcome and for the clinic-based outcomes (BP control and BP measurements), although we acknowledge substantial missingness in these clinic-based outcomes in that 32.7% of patients did not have a clinic visit during the study. We included all patients randomized as the denominator for patients with controlled BP, which likely underestimated actual control. To address the missing data, we carried out a multiple imputation. The study also included a large proportion of Black patients (86.1%), who typically have worse outcomes for hypertension, potentially helping to mitigate disparities.

The main limitation of the study was that the BP readings from routine visits may have been subject to selection factors or variation in measurement. This was due to the pragmatic nature of the study, which relied on routine clinical office visits for BP outcomes rather than dedicated research measurement; thus, we did not have BP data for about one-third of the patients.^26^ However, the proportion of patients with clinic BP readings was similar across arms, and we found similar results with multiple imputation. Additionally, the first tier of escalation for patients was to receive social support, but this proved unavailable to many patients who may have benefited from it. The study also relied on dedicated clinical staff for the intervention, which may be hard to generalize in other health systems. As this was a pragmatic study, we also did not have access to prescription fill and robust medication adherence data.

## Conclusions

This randomized clinical trial found that opt-out framing, by sending participants a BP monitor, was not more effective at increasing participation in remote BP management than opt-in framing, consisting of a recruitment letter and telephone calls to obtain consent before sending a BP monitor. However, the remote monitoring intervention overall improved BP control compared with usual care in the control arm. The findings suggest that future interventions are needed to make it easier for patients to participate in remote BP monitoring and to understand which components are most important for a successful program when implemented.

## Notes

Supplement 1.
Trial Protocol

Supplement 2. **eTable 1.** Characteristics of Consented Participants in Intervention Arms **eTable 2.** Average Number of Blood Pressure (BP) Readings Submitted by Arm **eTable 3.** Rate of Engagement in BP Monitoring by Arm (at Least 50% of BPs Submitted) **eTable 4.** Mean Diastolic Blood Pressure and Differences by Arm **eTable 5.** Patient Submitted Blood Pressures by Arm **eTable 6.** Patient Reported Medication Adherence by Arm

Supplement 3.
Data Sharing Statement
