# Supplementary material for: Behavioral Economic Framing for Enrollment and Retention of Patients in Remote Blood Pressure Monitoring: A Randomized Clinical Trial
Source: JAMA Netw Open. 2025 Sep 2;8(9):e2529825. doi: 10.1001/jamanetworkopen.2025.29825 (PMC12406065; doi:10.1001/jamanetworkopen.2025.29825)
Supplement: Supplement 1. — Trial Protocol [file jamanetwopen-e2529825-s001.pdf]

Supplement

SupportBP 2.0: Behavioral Science and Enrollment in Remote Monitoring for Hypertension Management

This supplement provides additional information about the work. It contains the following items:

Initial Protocol ……… 1

Final Protocol ……… 15

Summary of protocol changes ………………………………………………………………………………………………….. 30

Appendix A: Final Recruitment Materials……………………………………………………………………………………… 31

Appendix B: Final Text Messaging Content...………………………………………………………………………………….. 35

Appendix C: Stepped Escalation Intervention Flow Diagram ……………………………………………………………… 51

Appendix D: Final Interview Guide …. ………………………………………………………………………………………… 52

Initial Protocol

SupportBP 2.0: Behavioral Science and Enrollment in Remote Monitoring for Hypertension Management

Abstract
A 3-arm randomized pilot trial aimed at comparing the effectiveness of different approaches to recruiting patients to participate in a 6-month remote monitoring program for the management of hypertension (HTN). 425 eligible patients will be randomized in a 2:2:1 ratio into three recruitment arms: A) Opt-in Recruitment (mailed letter + recruitment phone call), B) Opt-out Recruitment (mailed blood pressure cuff + letter with opt-out messaging + recruitment phone call), or C) Usual Care (no contact). The targeted population is patients aged 18-75 within the Penn Family Care practice with at least 1 in-person office visit within the prior 12 months and the last blood pressure reading exceeding recommended HTN guidelines.

Study Instruments
A subset of 50 participants in the monitoring arms will be randomly selected to complete a post-program interview. The post-program interview will collect adherence and monitoring information, as well as qualitative data regarding participant perceptions about the intervention.

Group Modifications
Patients in the usual care arm will not be contacted by study staff, they will not receive a blood pressure cuff, and they will not receive any text messaging or any component of the stepped program. Patients assigned to recruitment arm A (opt-in) will receive a letter describing the program and inviting them to participate followed by a recruitment phone call requesting participation. If the patient consents, the research coordinator will mail a blood pressure cuff and proper measurement instructions, and start their remote monitoring program in the Way to Health platform. Patients will also be asked to provide the names of up to 3 friends or family members to serve as a potential support partner. Patients assigned to recruitment arm B (opt-out) will receive a blood pressure cuff with instructions in the mail, along with a letter describing the program with opt-out framing regarding their participation. Patients in this arm will also receive a recruitment phone call similar to recruitment arm A, requesting participation and the names of up to 3 potential support partners. If the patient consents, the research coordinator will start their remote monitoring program in the Way to Health platform. Participants in both groups will proceed through the same remote monitoring program regardless of recruitment method, and participants of either group may end their participation at any time.

Administration of Surveys
The post-program interview will be conducted by phone with 50 randomly selected participants assigned to one of the monitoring arms and will collect adherence and monitoring information, as well as qualitative data regarding patient perceptions about the monitoring program. We anticipate the interview to take no longer than 15-20 minutes to complete. Patients will verbally consent to the interview and to recording of the interview. Research staff will make no more than three attempts to speak directly with the participant.

Objectives
<u>1.1 Objectives</u>
We plan to evaluate the effectiveness of an opt-out approach to recruitment into a remote monitoring program for the management of hypertension, versus the more conventional opt-in model of recruitment. We will utilize the Way to Health (WTH) platform to develop and evaluate a new remote monitoring

enrollment model that is integrated with routine clinical care at Penn Family Care (PFC), an academic family medicine practice in West Philadelphia. Among patients with poorly controlled HTN:

1. We will compare the effect of sending eligible patients a blood pressure cuff with the mailed invitation letter and opt-out framing versus the more conventional method of phone calls following mailed letters.
2. We will also evaluate the feasibility and effectiveness of a remote monitoring program with individualized stepped escalation for hypertension management.

1.2 Primary Outcome Variable

The primary outcome is the proportion of patients participating in each study arm out of the number randomized to that study arm.

1.3 Secondary Outcome Variable(s)

Secondary outcomes include the proportion of requested BP measurements submitted by each patient by study arm, the proportion of patients recruited by each method who are actively engaged with the program (defined as submitting at least 50% of the requested BP measurements over the duration of the program), the proportion with controlled blood pressure by remote monitoring and the proportion with controlled blood pressure by clinic visits (usual care). We will additionally evaluate the systolic and diastolic blood pressure trajectory by recruitment arm. Lastly, we will gather qualitative insights from patients and providers about their experience with and perception of both the recruitment and remote monitoring programs.

Additional outcomes include an evaluation of the escalation criteria, escalation frequency, and text message content value.

Background

Remote monitoring has shown improvement in hypertension (HTN) control, but the benefits of such programs have not been sustained in a scalable way. There is opportunity to develop automated approaches to monitoring blood pressure and medication by leveraging behavioral economics and clinical practice redesign. There is also an imperative at Penn Medicine to reduce face-to-face visits and manage hypertension virtually as a result of the COVID-19 pandemic.

Prior work has shown that reported medication adherence as measured by bidirectional texting or electronic pill bottles was high, but was not sufficient on its own for improving blood pressure control. Additional work in this area has taught us that even using exception handling for patient data, PCPs did not have the capacity to effectively manage out of range blood pressure (BP) readings, and a centralized, dedicated NP/RN was created at Penn Family Care to manage this work. The Primary Care Service Line is also working on identifying a remote management pathway and have identified Care Managers as primarily responsible for the escalation of patients to PCPs for titration and med adjustments as needed, but this too is plagued with difficulties in recruitment and clinical inertia.

Ultimately, none of this prior work was has been sufficient to control BP in a sustainable manner, but different components of all of these projects have shown promise in feasibility, engagement, clinical workflow, and a non-significant but potentially clinically relevant improvement in BP.

In a remote management context, patient participation and engagement with the program are key even before we can begin to evaluate effectiveness of the remote intervention. We are proposing to evaluate both different strategies to recruit and engage patients with the remote monitoring program, as well as the overall effectiveness and feasibility of the clinical workflow created by the program itself.

Statistical Considerations

1.1 Power and sample size

Based on a previous pilot in the same clinical population, we estimate that 31% of eligible patients offered the remote monitoring program will agree to participate in the opt-in arm. We plan to enroll 425 patients in the study in a 2:2:1 ratio, with 170 in each recruitment arm, and 85 in the usual care arm. Considering a two-sided type I error rate of .05, we have 80% power to detect an increase in recruitment of 15%, with opt-out compared to opt-in enrollment. This would be clinically significant and feasible based on prior opt-out studies we have conducted.

Irrespective of whether the differences between arms achieve statistical significance, the study will provide valuable information about recruitment rates that can be used to guide study design, and particularly recruitment goals, for future clinical trials. Table 1 shows that if the observed recruitment rate for the study is 30% we can with high confidence assume that the actual rate in the overall population lies between 24 and 36%. In contrast if the observed rated is 45%, we can, with high confidence, assume that the actual rate in the overall population is considerably higher, between 39 and 51%. This study can also provide estimates of differences in the enrollment rates between recruitment methods. For example, Table 2 shows that the observed rate difference is 15% (for individual group rates of 30-45%), then the 90% CI suggests that the actual rates in the population lie between 6-24%. This information could prove valuable in assessing for example cost effectiveness of the proposed opt-out strategy.

Table 1: Estimation of enrollment rates for individual recruitment strategies for two-sided 90% CI (n=170 per group)

Observed Rate (%)	½ width of 90% CI (Margin of error %)	Endpoints of 90% CI (%)
10	3.8	(6.8, 14.4)
20	5.0	(15.4,25.5)
**30**	**5.7**	**(24.6, 36.1)**
40	6.1	(34.0, 46.3)
**45**	**6.2**	**(38.9 51.3)**

Table2: Estimation of enrollment differences with two-sided 90% CI (170 per group)

Observed Rates (%)	Rate difference (%)	Rate Ratio	½ width of 90% CI (Margin of error)	Endpoints of 90% CI (%)
10-20	10	2.0	6.9	(3.1, 16.9)
10-25	15	2.5	7.2	(7.8, 22.2)
20-30	10	1.5	7.7	(1.7, 18.3)
20-35	15	1.75	8.8	(6.6, 23.4)
30-40	10	1.33	9.0	(1.0, 19.0)
**30-45**	**15**	**1.50**	**9.1**	**(5.8, 24.1)**

Lastly, we conservatively anticipate an SD of around 12 (DBP) to 15 (SBP) for the BP measurements resulting in excellent precision to estimate the mean BP post-intervention in each of the groups. Conservatively, assuming that 30% of the randomized sample ultimately have usable blood pressure measurements and a randomized sample size of 170 per arm, the precision (margin of error) of the two-sided 90% CI on mean

DBP will be +/- 2.3 mmHg and for SBP will be +/- 3.5 mmHg. These calculations are for the individual arms. The difference in means for the two arms will be estimated to +/- 4.6 mmHg for DBP and to +/-6.9 mmHg for SBP. If a larger number of subjects have BP measurements the precision of these estimates will be improved.

1.2 Data analysis

The data, including the outcome data, baseline demographics and clinical characteristics, will be explored, overall and by arm, using graphical tools and summary statistics. The primary analysis will compare the proportion of patients participating in each study arm using a Z-test. We will calculate estimates and 90% confidence intervals (CI) for the rates of enrollment for each group and the difference in rates and the 90% CI on the difference. Using a logistic regression model, we will explore whether participation in an arm differs by baseline demographic and clinical characteristics

We will also compare the mean rates of participation in BP measurements in the two arms using a generalized estimating equations (GEE) model that includes the number of BP measurements submitted by each patient with a separate offset for the number of measurements requested. Because participation in the arms may differ by demographic/clinical characteristics, we will secondarily adjust the model for these variables. Similarly, using a logistic regression model, we will estimate the odds of engagement (defined as submitting at least 50% of the requested BP measurements over the duration of the program) between recruitment arms, again with adjustment for demographic and clinical characteristics. Similarly we will estimate the total proportion with controlled BP in the program arms and usual care arm. We will quantify the mean, standard deviation (SD), median and interquartile range of the BP measurements at baseline and at the conclusion of the study. Using a linear model, we will determine whether mean BP differs by arm after adjustment for baseline BP and use the model to provide estimates of both final BP as well as changes in blood pressure from baseline. Final blood pressure measurements for home monitoring at end of study will be the average of the final 3 patient submitted blood pressures for those enrolled in the program. We will use the blood pressure measurement(s) from the most recent in-person clinic visit for all arms.

Additional outcomes include an evaluation of the escalation criteria, escalation frequency, and text message content value.

Study Design

1.1 Design

This is randomized control trial of recruitment methodologies with approximately 425 patients being recruited to participate in a 6 month escalating, stepped remote monitoring program to control blood pressure. Eligible participants will be randomized in a 2:2:1 ratio to three recruitment arms in variable blocks of 5 and 10 using a computer-generated randomization algorithm: A) opt-in recruitment (mailed letter + recruitment phone call), B) opt-out recruitment (mailed blood pressure cuff and letter with opt-out messaging + recruitment phone call), or C) usual care (no contact). Using the Way to Health (WTH) platform for bidirectional text messaging with patients, we will iterate on the design of the program and implement learnings at 6-8 week intervals to identify best methods for participant engagement, messaging content, escalation criteria, escalation frequency, titration period and order of steps.

1.2 Consent Process

We are requesting a waiver of the requirement to document consent and HIPAA authorization with a signature for participants randomized into a recruitment arm for this study, since we believe the research presents no more than minimal risk of harm to subjects and involves no procedures for which written consent is normally required outside of the research context. All participants being enrolled in this study

will be enrolled via a remote recruitment process, therefore we will read the IRB approved Consent/HIPAA script over the phone to each patient and ask them to provide verbal consent and HIPAA authorization for use of their data in the study. A copy of the consent form will also be mailed to the participant's home address for their review prior to receiving any recruitment calls from research staff. As all recruitment will be done over the phone, and as there are no in-person visits required as part of this study, a waiver of the requirement to document consent and HIPAA authorization with a signature is requested.

Participants who are randomized to the control arm will not be recruited for participation in the remote monitoring program. We request a waiver of consent for participants randomized to this study arm, as the consent process itself may influence the study outcomes, we are requesting a waiver of consent since we believe the research presents no more than minimal risk of harm to this group of participants.
The remote monitoring program has been designed in collaboration with Penn Family Care clinicians and the program is clinically appropriate for those with uncontrolled hypertension. All participants will continue to receive standard clinical care. All participants randomized to the recruitment arms will also be informed that this program is voluntary and that they can stop participating at any time.

Study Duration
We expect recruitment to take approximately 5 months. The program itself will be 6 months from the date the participant agrees to participate, and we will need an additional 3 months to complete qualitative interviews with a random sample of 50 participants, as well as clinicians from Penn Family Care. Including data analysis and manuscript preparation, total study duration should not exceed 15 months.

Resources Necessary for Human Research Protections
Dr. Mehta along with Project Manager (Catherine Reitz) and Clinical Research Coordinators (Lauren Ianotte, Kiernan McNelis, and Caitlin McDonald) are adequately informed of the protocol and adequately qualified to conduct research via training required for medical doctors/students and research coordinators. All are up to date with HIPAA and CITI training.

Detailed Standard Operating Procedure documents for the project will be accessible to all members of the research team, which will keep research staff informed about the protocol and their related duties. There are adequate facilities to conduct the research.

Target Population
The targeted population is patients aged 18-75 within the Penn Family Care practice with at least 1 in-person office visit within the prior 12 months and the last blood pressure reading exceeding recommended HTN guidelines.

Subjects Enrolled by Penn Researchers
425

Subjects Enrolled by Collaborating Researchers
0

Accrual
Participants in this study will be identified via electronic medical records review by our study staff. The study staff will work with UPHS to create a filter for the electronic medical record system that will generate a list of patients who may meet the study criteria. A study coordinator will review the list of

patients to determine eligibility. If patients meet the minimal requirements they will be randomized and recruited according to study arm. Based on a previous pilot in the same clinical population, we estimate that 31% of eligible patients offered the remote monitoring program will agree to participate in the opt-in recruitment arm. We plan to enroll 425 patients in the study in a 2:2:1 ratio, with 170 in each recruitment arm, and 85 in the usual care/no contact arm. Considering a two-sided type I error rate of .05, we have 80% power to detect an increase in recruitment of 15%, with opt-out compared to opt-in enrollment. Recruitment will end once 425 eligible participants have been randomized.

Key Inclusion Criteria

1. Between 18 and 75 years old with HTN (ICD-10 code I10)
2. Has had at least one office visit at Penn Family Care (PFC) within the past 12 months (at time of chart review), with the last visit having a BP reading exceeding HTN guidelines (150/90 if ≥60 or, 140/90 if ages 21-59 yrs or has CKD or diabetes).
3. Must have a cellular phone with texting capabilities
4. Must be prescribed at least one medication for hypertension

Key Exclusion Criteria

1. Has metastatic (Stage IV) cancer
2. Has end stage renal disease
3. Has congestive heart failure
4. Has dementia
5. BMI >= 50
6. Is Non-English speaking requiring a translator

Vulnerable Populations

No vulnerable populations are specifically included or excluded in the research study. We will include all eligible patients in the Penn Family Care practice.

Populations Vulnerable to Undue Influence or Coercion

We are not specifically targeting any vulnerable populations.

Subject Recruitment

Participants in this study will be identified via electronic medical records review by our study staff. The study staff will work with UPHS to create a filter for the electronic medical record system that will generate a list of patients who may meet the study criteria. A study coordinator will review the list of patients to determine eligibility. If patients meet the minimal requirements they will be randomized to three recruitment arms in variable blocks of 5 and 10 using a computer-generated algorithm, and recruited according to study arm. Recruitment will end once 425 eligible participants have been randomized and all contact attempts exhausted.

Up to three potential support partners will be identified by the participant at the time of their enrollment. If the participant reports at least 3 of 10 elevated blood pressure measurements (as defined by guidelines), the CRC will make up to 3 attempts to contact and enroll the first support partner provided. If they decline or are not reachable, the CRC will make up to 3 call attempts each with the second and third support partners until a partner has been enrolled or until all call attempts have been exhausted with all partners provided. Support partners will be informed of the research the participant is part of, and offer their verbal agreement to participate in the program.

Subject Compensation
Participants will not be compensated for their participation.

Procedures
Recruitment: A data pull from Clarity will be used to identify potentially eligible patients at the Penn Family Care family medicine practice. Eligible patients will have a diagnosis of HTN, be taking at least one BP medication, have a valid cell phone and text messaging plan, and have an elevated BP measurement per guidelines within the last year. Patients will be excluded if they have a history of CHF, dementia, BMI >=50, ESRD or metastatic cancer. In variable blocks of 5 and 10 using a computer-generated algorithm, eligible patients will be randomized into three recruitment groups: A) opt-in recruitment (mailed letter + recruitment phone call), B) opt-out recruitment (mailed blood pressure cuff + letter with opt-out messaging + recruitment phone call), or C) usual care (no contact).

Patients in the usual care arm will not be contacted by study staff, they will not receive a blood pressure cuff, and they will not receive any text messaging or any component of the stepped program.

Patients in the opt-in recruitment arm will receive a letter describing the program and inviting them to participate. Along with the letter they will receive an informational brochure and a copy of the informed consent form. Patients in this arm will then receive a recruitment phone call explaining the program and consenting the participant verbally. If the participant consents to participate in the remote monitoring program, to receive text messaging, and to connection to care, the research coordinator will mail a blood pressure cuff and begin the remote monitoring program for the patient in the Way to Health platform. Patients will also be asked to provide the names of up to 3 friends or family members to serve as a potential support partner and to confirm correct BP cuff sizing.

Patients assigned to opt-out recruitment will receive a blood pressure cuff with instructions in the mail along with a letter describing the program and opt-out framing regarding their participation, as well as a brochure and a copy of the informed consent form. Patients in this arm will receive a recruitment phone call similar to recruitment arm A, explaining the program and consenting the participant verbally. If the participant consents to participate in the remote monitoring program, to receive text messaging, and to connection to care, the research coordinator will begin the remote monitoring program for the patient in the Way to Health platform. Patients will also be asked to provide the names of up to 3 potential support partners and to confirm correct BP cuff sizing.

For both the Opt-In and Opt-Out recruitment arms, the Research Coordinators will make up to 3 phone call attempts to reach the patient. If the patient cannot be contacted after 3 attempts, they will be marked as unreachable and no further attempts to recruit the patient will be made.

Participants in both recruitment groups will proceed through the same remote monitoring program regardless of recruitment method and in collaboration with their care team at the Penn Family Care practice. Participants of either group may end their participation in the program at any time. Participants of either recruitment group who are found to be unreachable will be mailed a debrief letter explaining their participation in the program.

Remote Monitoring Program: All patients will begin the program in the "Enhanced Baseline" phase and will escalate through the 5 program steps only as needed for BP control. All patient submitted BP measurements will be visible to clinicians from the WTH integrated BP flowsheet. Each step of the intervention and escalation criteria are described below (also see attached flowsheet).

Step 1 - Enhanced Baseline: patients will receive BP prompts three times each week ("What is your blood pressure today?"), a medication adherence prompts ("Have you taken your [drug name] as prescribed this week?"), positive feedback on BP measurements if controlled ("You've submitted three controlled measurements in a row! Great job!"), lifestyle messaging (diet, exercise, self-efficacy, and other tips relevant for controlling hypertension), and feedback and notice of escalation to the nursing pool ("You've submitted three elevated measurements in a row, we will notify your Care Team and step up your intervention"). If the patient submits any 3 elevated readings (per guidelines) in a 3-week period, we will notify the nursing pool consisting of at least one designated nurse (RN) and one Nurse Practitioner (NP), and step the patient to the next level of the program, otherwise they will remain in this step through the end of their 6-month period.

Severely elevated readings (≥180/110) will receive an automated response informing them that the reading is high and request the measurement be taken again and resubmitted after 15 minutes. Patients will further be informed to contact the practice if it remains elevated. After the second reading is obtained, both measurements will be routed to the nursing pool for evaluation. If no response is re-submitted after 15 minutes, a prompt will be sent to the patient to resubmit, along with instructions to contact the practice if they are experiencing symptoms; the initial measurement will be routed alone.

Step 2 - Enhanced Cheerleading: If the participant reports at least 3 of 10 elevated blood pressure measurements (as defined by guidelines), the CRC will make up to 3 attempts to contact and enroll the first support partner provided during the recruitment phone call. If the first support partner declines or is not reachable, the CRC will make up to 3 call attempts each with the second and third support partners until a partner has been enrolled or until all call attempts have been exhausted with all partners provided. Patients will continue to receive all components of the baseline program and the patient will be notified that their support partner has been added. The support partner will receive BP measurement and adherence information submitted by the patient and encouraged to engage with the patient in personal conversation about his/her health ("[Patient Name] is struggling to keep their BP controlled, talk to them about how important their health is to you"). Positive feedback and milestones achieved by the patient will also be shared ("[Patient name has submitted 3 controlled BP measurements in a row, we'll let them know how proud you are unless you reply "N" to this message in the next 2 hours"), and messages will be personalized with names and relationships to the extent possible. If the patient submits 3 elevated readings (per guidelines) in a 3-week period, we will notify the nursing pool and step the patient to the next level of the program, otherwise they will remain in this step through the end of their 6-month period.

Step 3 - Clinical Two-Way Texting: patients will continue to receive all components of the baseline program and enhanced cheerleading, and will additionally receive asynchronous text communication with the nursing pool for coaching and clarity related to medication adherence, symptoms, and/or any lifestyle factors that may need adjustment prior to medication titration. The nursing pool will be able to access the SMS inbox for each patient directly from the patient record. If the patient 3 elevated readings (per guidelines) in a 3-week period, we will notify the nursing pool and step the patient to the next level of the program, otherwise they will remain in this step through the end of their 6-month period. Asynchronous texting with the nursing pool may not continue if coaching is successful and BP is successfully controlled at this step.

Step 4 - Titration: patients will continue to receive all components of the three previous steps. The designated Nurse Practitioner will review patient submitted BP measurements through the integrated

flowsheet, results at each step of the program and any information gathered through Clinical 2-Way Texting to make dosing or medication changes or add additional medications for BP control. Once the medication change has been implemented, adoption and adherence will be confirmed through Clinical 2-Way texting. If after a 4-week adoption period the patient again submits at least 3 elevated readings (per guidelines) in a 3-week period, we will notify the nursing pool and step the patient to the next level of the program, otherwise they will remain in this step through the end of their 6-month period.

Step 5 - Office/Telemedicine Visit: patients will continue to receive all components of the four previous steps. The Nurse Practitioner will meet with the patient in the office or via a virtual/telemedicine visit for additional clinical workup, referral, or additional medication changes. The patient will have this visit and remain in this phase through the end of their 6-month period. If the patient 3 elevated readings (per guidelines) in a 3-week period, we will notify nursing pool and they will, at their discretion, implement additional medication changes, see the patient again, or refer for additional clinical workup elsewhere.

The nursing pool will be notified of all escalations. At any point of escalation to the next step, the RN or NP may choose to directly escalate to step 4 or Step 5 for titration or a visit at his or her discretion, skipping any steps in between as deemed appropriate.

Assessment/Iteration: In 6-8 week intervals, we plan to assess the number of patients who are actively engaged with the program, the number of patients at each step, the percent controlled at each step, and the number of patients skipped directly to Step 4 or Step 5 by a member of the Nursing Pool. We will also evaluate participant engagement, escalation criteria, escalation frequency, and text message content value and adjust as needed to improve BP control among our participants.

Qualitative Assessment: We will interview a random subset of 50 patients to assess final patient engagement and gather additional qualitative insights about their experience and perception with the program. We anticipate these interviews to take no more than 15-20 minutes by phone. Research staff will make no more than three attempts to speak directly with the subject. Participants will be informed the interviews will be recorded and transcribed and must agree to participate in the interview before it begins. Their agreement will be recorded in REDCap. Similarly, we will interview the clinicians participating in the Nursing Pool and a random set of PCPs whose patients participated in the program, to assess their experience and perception of the program.

Analysis Plan

Irrespective of whether the differences between arms achieve statistical significance, the study will provide valuable information about recruitment rates that can be used to guide study design, and particularly recruitment goals, for future clinical trials. Table 1 shows that if the observed recruitment rate for the study is 30% we can with high confidence assume that the actual rate in the overall population lies between 24 and 36%. In contrast if the observed rated is 45%, we can, with high confidence, assume that the actual rate in the overall population is considerably higher, between 39 and 51%. This study can also provide estimates of differences in the enrollment rates between recruitment methods. For example, Table 2 shows that the observed rate difference is 15% (for individual group rates of 30-45%), then the 90% CI suggests that the actual rates in the population lie between 6-24%. This information could prove valuable in assessing for example cost effectiveness of the proposed opt-out strategy.

Table 1: Estimation of enrollment rates for individual

recruitment strategies for two-sided 90% CI (n=170 per group)		
Observed Rate (%)	½ width of 90% CI (Margin of error %)	Endpoints of 90% CI (%)
10	3.8	(6.8, 14.4)
20	5.0	(15.4,25.5)
**30**	**5.7**	**(24.6, 36.1)**
40	6.1	(34.0, 46.3)
**45**	**6.2**	**(38.9 51.3)**

Table2: Estimation of enrollment differences with two-sided 90% CI (170 per group)				
Observed Rates (%)	Rate difference (%)	Rate Ratio	½ width of 90% CI (Margin of error)	Endpoints of 90% CI (%)
10-20	10	2.0	6.9	(3.1, 16.9)
10-25	15	2.5	7.2	(7.8, 22.2)
20-30	10	1.5	7.7	(1.7, 18.3)
20-35	15	1.75	8.8	(6.6, 23.4)
30-40	10	1.33	9.0	(1.0, 19.0)
**30-45**	**15**	**1.50**	**9.1**	**(5.8, 24.1)**

Lastly, we conservatively anticipate an SD of around 12 (DBP) to 15 (SBP) for the BP measurements resulting in excellent precision to estimate the mean BP post-intervention in each of the groups. Conservatively, assuming that 30% of the randomized sample ultimately have usable blood pressure measurements and a randomized sample size of 170 per arm, the precision (margin of error) of the two-sided 90% CI on mean DBP will be +/- 2.3 mmHg and for SBP will be +/- 3.5 mmHg. These calculations are for the individual arms. The difference in means for the two arms will be estimated to +/- 4.6 mmHg for DBP and to +/-6.9 mmHg for SBP. If a larger number of subjects have BP measurements the precision of these estimates will be improved.

1.2 Data analysis

The data, including the outcome data, baseline demographics and clinical characteristics, will be explored, overall and by arm, using graphical tools and summary statistics. The primary analysis will compare the proportion of patients participating in each study arm using a Z-test. We will calculate estimates and 90% confidence intervals (CI) for the rates of enrollment for each group and the difference in rates and the 90% CI on the difference. Using a logistic regression model, we will explore whether participation in an arm differs by baseline demographic and clinical characteristics

We will also compare the mean rates of participation in BP measurements in the two arms using a generalized estimating equations (GEE) model that includes the number of BP measurements submitted by each patient with a separate offset for the number of measurements requested. Because participation in the arms may differ by demographic/clinical characteristics, we will secondarily adjust the model for these variables. Similarly, using a logistic regression model, we will estimate the odds of engagement (defined as submitting at least 50% of the requested BP measurements over the duration of the program) between recruitment arms, again with adjustment for demographic and clinical characteristics. Similarly we will estimate the total proportion with controlled BP in the program arms and usual care arm. We will

quantify the mean, standard deviation (SD), median and interquartile range of the BP measurements at baseline and at the conclusion of the study. Using a linear model, we will determine whether mean BP differs by arm after adjustment for baseline BP and use the model to provide estimates of both final BP as well as changes in blood pressure from baseline. Final blood pressure measurements for home monitoring at end of study will be the average of the final 3 patient submitted blood pressures for those enrolled in the program. We will use the blood pressure measurement(s) from the most recent in-person clinic visit for all arms.

Additional outcomes include an evaluation of the escalation criteria, escalation frequency, and text message content value.

Data Confidentiality
Paper-based records will be kept in a secure location and only be accessible to personnel involved in the study. Computer-based files will only be made available to personnel involved in the study through the use of access privileges and passwords. Prior to access to any study-related information, personnel will be required to sign statements agreeing to protect the security and confidentiality of identifiable information. Wherever feasible, identifiers will be removed from study-related information. Precautions are in place to ensure the data is secure by using passwords and encryption, because the research involves web-based surveys. Audio and/or video recordings will be transcribed and then destroyed to eliminate audible identification of subjects.

Subject Confidentiality
Information about study subjects will be kept confidential and managed according to the requirements of the Health Insurance Portability and Accountability Act of 1996 (HIPAA). All PHI will be maintained on UPHS servers. Source documents are maintained in PennChart. No source documents will be printed or maintained in paper form at the study site. Data from PennChart will be recorded in Penn Medicine's REDCap system. The investigator and study team (which includes the project manager and research coordinators) will have access to PHI within PennChart and REDCap. We will label all PHI within REDCap as identifiable information so that de-identified exports are possible. All reports that include identifiable information will be stored on the Innovation Center secure drive, maintained behind the UPHS firewall. Once data analysis and manuscripts have been published, the databases will be removed from REDCap and the data will be de-identified on the secure drive. This de- identified dataset will be stored for up to five years after analysis is complete and manuscripts have been published. Once analysis is completed and any manuscripts are published, we will retain PHI no longer than seven years in accordance with government regulations, applicable policies, and institutional requirements. Phone calls will be transcribed using Datagain Transcription, a HIPAA compliant transcription service. Datagain's system controls, database architecture and internal policies provide HIPAA compliance.

Database Security/Protection Against Risk
Information about study subjects will be kept confidential and managed according to the requirements of the Health Insurance Portability and Accountability Act of 1996 (HIPAA). All PHI will be maintained on UPHS servers. Source documents are maintained in PennChart. No source documents will be printed or maintained in paper form at the study site. To assure that patient, physician and other informant confidentiality is preserved, individual identifiers (such as name and medical record number/physician billing identifier) are stored in a single password protected system that is accessible only to study research, analysis and IT staff. This system is hosted on site at The University of Pennsylvania (UPenn) and is protected by a secure firewall. Once a participant is in this system, they will be given a unique study

identification number (ID). Any datasets and computer files that leave the firewall will be stripped of all identifiers and individuals will be referred to by their study ID. The study ID will also be used on all analytical files.

The initial patient information collected for screening and recruitment will consist of name, address, phone number, demographic information such as race and income, and medication adherence practices. As our patient population is patients in the UPHS health system, the majority of this information will come from Electronic Chart reviews.

Sensitive Research Information

This research does not involve collection of sensitive information about the subjects that should be excluded from the electronic medical record.

Subject Privacy

We will only interact with the subsample of subjects with which we plan to enroll in the study. With these participants, we will conduct phone calls in a private area. When we call subjects, we will confirm the identify before beginning the recruitment process.

Data Disclosure

Blood pressure measurements will be disclosed to the designated centralized nursing pool within Penn Family Care, and may be provided to the primary care physician as deemed appropriate by the Nurse Practitioner, or if they reach or surpass a pre-established threshold determined to be an immediate danger to the subject's health.

Protected Health Information/Data Protection

- Name
- Street address, city, county, precinct, zip code, and equivalent geocodes
- All elements of dates (except year) for dates directly related to an individual and all ages over 89
- Telephone and fax numbers
- Medical record numbers
- Biometric identifiers, including finger and voice prints

Consent Process

<u>1.1 Overview</u>

We are requesting a waiver of the requirement to document consent and HIPAA authorization with a signature for participants randomized into a recruitment arm for this study, since we believe the reseach presents no more than minimal risk of harm to subjects and involves no procedures for which written consent is normally required outside of the research context. The remote monitoring program has been designed in collaboration with Penn Family Care clinicians and the program is clinically appropriate for those with uncontrolled hypertension. All participants will continue to receive standard clinical care. All participants being enrolled in this study will be enrolled via a remote recruitment process, therefore we will read the IRB approved Consent/HIPAA script over the phone to each patient and ask them to provide verbal consent and HIPAA authorization for use of their data in the study. A copy of the consent form will also be mailed to the participant’s home address for their review prior to receiving any recruitment calls from research staff. All participants randomized to the recruitment arms will also be informed that this

program is voluntary and that they can stop participating at any time. For these reasons, and because all recruitment will be done remotely and no in-person visits required as part of this study, a waiver of the requirement to document consent and HIPAA authorization with a signature is requested.

Participants who are randomized to the control arm will not be recruited for participation in the remote monitoring program. We request a waiver of consent for participants randomized to this study arm, as the consent process itself may influence study outcomes, because we believe the research presents no more than minimal risk of harm to participants.

Verbal consent will be obtained from the subsample with whom we plan to conduct post-intervention interviews. These subjects will be informed about the purpose of the phone call, asked if they would like to participate and if the phone call can be recorded. Since the interviews will be conducted by phone, verbal consent will be obtained and recorded in REDCap.

<u>1.2 Children and Adolescents</u>
Not applicable

<u>1.3 Adult Subjects Not Competent to Give Consent</u>
Waiver of consent is being requested.

Waiver of Consent
<u>1.1 Minimal Risk</u>
The research presents no more than minimal risk of harm to subjects and involves no procedures for which written consent is normally required outside of the research context. The research related activity is the randomization of subjects to different types of recruitment strategies.

<u>1.2 Impact on Subjects Rights and Welfare</u>
Participation in the remote monitoring program is completely voluntary and the participants rights and welfare will not be adversely affected by the waiver of documentation of consent.

<u>1.3 Waiver Essential to Research</u>
As the consent process itself may influence study outcomes we are requesting a waiver of consent for participants randomized to the control arm because we believe the research presents no more than minimal risk of harm to participants, and explaining the remote monitoring program to a participant in the Usual Care arm may influence the patient's behavior during the program period. We are requesting a waiver of the requirement to document consent and authorization with signature for participants enrolled in the intervention because we believe the research presents no more than minimal risk of harm to subjects and involves no procedures for which written consent is normally required outside of the research context.

<u>1.4 Written Statement of Research</u>
Upon completion of the study, we will send a letter to patients randomized to the remote monitoring arms that we are unable to reach for recruitment, explaining that we ran a quality improvement trial focused on enrollment and remote monitoring of blood pressure.

Potential Study Risks

As this study does not involve any medical decision making and only tests the use of behavioral approaches to encouraging patients to participate in a remote monitoring program for hypertension management, we consider this study minimal risk. The primary risk would be from a breach of confidentiality involving medical records reviews and monitoring of hypertension medication and blood pressure adherence with text messaging which will be maintained on the Way to Health platform. This risk has been mitigated by extensive privacy protection protocols, a highly secure data storage system, and a plan to remove identifiers from the data wherever possible. In addition, all personnel will be held to high standards of upholding confidentiality and safeguarding patient privacy.

Potential Study Benefits

The immediate benefits of this study for participants may include an improvement in adherence to behaviors and medications that have been proven to be effective in improving patient outcomes. It is possible that the benefits for many participants will be minimal. However, as mentioned, we believe the risks are also minimal. Knowledge gained from the study will assist in development of recruitment methods for remote monitoring interventions in other high risk patient populations in which non-adherence rates are high and blood pressure remains uncontrolled. The potential public health impact of a successful recruitment to an intervention to improve BP control is great and could reduce the number of deaths from health related outcomes in the United States each year.

Data and Safety Monitoring

The study PI will be responsible for monitoring the data and safety of the study as well as the program, and ensuring the ongoing quality and integrity of the research study. The investigator will permit study-related monitoring, audits and inspections by government regulatory authorities and University compliance officers.

Risk/Benefit Assessment

This study is designed to test recruitment strategies for enrollment in a remote monitoring program that incorporates many components of prior interventions that have previously demonstrated promise in feasibility, clinical workflow, and potentially relevant improvement in BP. We believe the combination of these approaches in this intervention will provide the research and public health communities with important information that can lead to broad generalizability in treating people at risk for the above mentioned chronic diseases and death nationally, as these types of programs could be set up by healthcare systems to be broadly utilized. However, successful recruitment and engagement of patients is critical to the success of the program itself, particularly in a remote management context. With minimal risks, the potential public health impact of a successful recruitment to a program to improve BP control is great and could reduce the number of deaths from health related outcomes in the United States each year.

Final Protocol

Increasing surveillance rates for hepatocellular carcinoma among cirrhotic patients

****New changes from initial protocol notated in red, parts removed from initial protocol notated in strikethrough**

Abstract

A 3-arm randomized pilot trial aimed at comparing the effectiveness of different approaches to recruiting patients to participate in a 6-month remote monitoring program for the management of hypertension (HTN). 425 eligible patients will be randomized in a 2:2:1 ratio into three recruitment arms: A) Opt-in Recruitment (mailed letter + recruitment phone call), B) Opt-out Recruitment (mailed blood pressure cuff + letter with opt-out messaging + recruitment phone call), or C) Usual Care (no contact). The targeted population is patients aged 18-75 within the Penn Family Care practice with at least 1 in-person office visit within the prior 12 months and the last blood pressure reading exceeding recommended HTN guidelines.

Study Instruments

Participants who agreed to participate in the remote monitoring program will be assigned to participation groups based on the number and timing of blood pressure measurements submitted to the program, as well as on their engagement with the clinician performing patient follow-up if blood pressures were escalated for not being controlled. Within each of these participation groups, participants will be randomly selected for an interview to collect qualitative data regarding their level of participation, barriers or supports to participation, perceptions about the program and stepped levels of care, and opportunities for improvement in the remote monitoring program. Rather than pre-specify a number of patients that must be interviewed per group, the research team will be attentive to thematic saturation and use this as a guide that a sufficient number of interviews has been reached.

~~A subset of 50 participants in the monitoring arms will be randomly selected to complete a post-program interview. The post-program interview will collect adherence and monitoring information, as well as qualitative data regarding participant perceptions about the intervention.~~

Group Modifications

Patients in the usual care arm will not be contacted by study staff, they will not receive a blood pressure cuff, and they will not receive any text messaging or any component of the stepped program. Patients assigned to recruitment arm A (opt-in) will receive a letter describing the program and inviting them to participate followed by a recruitment phone call requesting participation. If the patient consents, the research coordinator will mail a blood pressure cuff and proper measurement instructions, and start their remote monitoring program in the Way to Health platform. Patients will also be asked to provide the names of up to 3 friends or family members to serve as a potential support partner. Patients assigned to recruitment arm B (opt-out) will receive a blood pressure cuff with instructions in the mail, along with a letter describing the program with opt-out framing regarding their participation. Patients in this arm will also receive a recruitment phone call similar to recruitment arm A, requesting participation and the names of up to 3 potential support partners. If the patient consents, the research coordinator will start their remote monitoring program in the Way to Health platform. Participants in both groups will proceed through the same remote monitoring program regardless of recruitment method, and participants of either group may end their participation at any time.

Administration of Surveys

The post-program interview will be conducted by phone ~~with 50~~ randomly selected participants assigned to one of the monitoring arms and to a participation group, and will collect ~~adherence and monitoring information, as well as~~ qualitative data regarding their level of participation, barriers or supports to participation, perceptions about the program and stepped levels of care, and opportunities for improvement within the remote monitoring program. ~~patient perceptions about the monitoring program.~~ We anticipate the interview to take no longer than ~~15-20~~20-30 minutes to complete. Patients will verbally consent to the interview and to recording of the interview. Research staff will make no more than three attempts to speak directly with the participant.

Objectives

1.4 Objectives

We plan to evaluate the effectiveness of an opt-out approach to recruitment into a remote monitoring program for the management of hypertension, versus the more conventional opt-in model of recruitment. We will utilize the Way to Health (WTH) platform to develop and evaluate a new remote monitoring enrollment model that is integrated with routine clinical care at Penn Family Care (PFC), an academic family medicine practice in West Philadelphia. Among patients with poorly controlled HTN:

3. We will compare the effect of sending eligible patients a blood pressure cuff with the mailed invitation letter and opt-out framing versus the more conventional method of phone calls following mailed letters.
4. We will also evaluate the feasibility and effectiveness of a remote monitoring program with individualized stepped escalation for hypertension management.

1.5 Primary Outcome Variable

The primary outcome is the proportion of patients participating in each study arm out of the number randomized to that study arm.

1.6 Secondary Outcome Variable(s)

Secondary outcomes include the proportion of requested BP measurements submitted by each patient by study arm, the proportion of patients recruited by each method who are actively engaged with the program (defined as submitting at least 50% of the requested BP measurements over the duration of the program), the proportion with controlled blood pressure by remote monitoring and the proportion with controlled blood pressure by clinic visits (usual care). We will additionally evaluate the systolic and diastolic blood pressure trajectory by recruitment arm. Lastly, we will gather qualitative insights from patients and providers about their experience with and perception of both the recruitment and remote monitoring programs.

Additional outcomes include an evaluation of the escalation criteria, escalation frequency, and text message content value.

Background

Remote monitoring has shown improvement in hypertension (HTN) control, but the benefits of such programs have not been sustained in a scalable way. There is opportunity to develop automated approaches to monitoring blood pressure and medication by leveraging behavioral economics and clinical practice redesign. There is also an imperative at Penn Medicine to reduce face-to-face visits and manage hypertension virtually as a result of the COVID-19 pandemic.

Prior work has shown that reported medication adherence as measured by bidirectional texting or electronic pill bottles was high, but was not sufficient on its own for improving blood pressure control.

Additional work in this area has taught us that even using exception handling for patient data, PCPs did not have the capacity to effectively manage out of range blood pressure (BP) readings, and a centralized, dedicated NP/RN was created at Penn Family Care to manage this work. The Primary Care Service Line is also working on identifying a remote management pathway and have identified Care Managers as primarily responsible for the escalation of patients to PCPs for titration and med adjustments as needed, but this too is plagued with difficulties in recruitment and clinical inertia.

Ultimately, none of this prior work was has been sufficient to control BP in a sustainable manner, but different components of all of these projects have shown promise in feasibility, engagement, clinical workflow, and a non-significant but potentially clinically relevant improvement in BP.

In a remote management context, patient participation and engagement with the program are key even before we can begin to evaluate effectiveness of the remote intervention. We are proposing to evaluate both different strategies to recruit and engage patients with the remote monitoring program, as well as the overall effectiveness and feasibility of the clinical workflow created by the program itself.

Statistical Considerations

<u>1.1 Power and sample size</u>

Based on a previous pilot in the same clinical population, we estimate that 31% of eligible patients offered the remote monitoring program will agree to participate in the opt-in arm. We plan to enroll 425 patients in the study in a 2:2:1 ratio, with 170 in each recruitment arm, and 85 in the usual care arm. Considering a two-sided type I error rate of .05, we have 80% power to detect an increase in recruitment of 15%, with opt-out compared to opt-in enrollment. This would be clinically significant and feasible based on prior opt-out studies we have conducted.

Irrespective of whether the differences between arms achieve statistical significance, the study will provide valuable information about recruitment rates that can be used to guide study design, and particularly recruitment goals, for future clinical trials. Table 1 shows that if the observed recruitment rate for the study is 30% we can with high confidence assume that the actual rate in the overall population lies between 24 and 36%. In contrast if the observed rated is 45%, we can, with high confidence, assume that the actual rate in the overall population is considerably higher, between 39 and 51%. This study can also provide estimates of differences in the enrollment rates between recruitment methods. For example, Table 2 shows that the observed rate difference is 15% (for individual group rates of 30-45%), then the 90% CI suggests that the actual rates in the population lie between 6-24%. This information could prove valuable in assessing for example cost effectiveness of the proposed opt-out strategy.

Table 1: Estimation of enrollment rates for individual recruitment strategies for two-sided 90% CI (n=170 per group)

Observed Rate (%)	½ width of 90% CI (Margin of error %)	Endpoints of 90% CI (%)
10	3.8	(6.8, 14.4)
20	5.0	(15.4,25.5)
**30**	**5.7**	**(24.6, 36.1)**
40	6.1	(34.0, 46.3)
**45**	**6.2**	**(38.9 51.3)**

Table2: Estimation of enrollment differences with two-sided 90% CI (170 per group)

Observed Rates (%)	Rate difference (%)	Rate Ratio	½ width of 90% CI (Margin of error)	Endpoints of 90% CI (%)
10-20	10	2.0	6.9	(3.1, 16.9)
10-25	15	2.5	7.2	(7.8, 22.2)
20-30	10	1.5	7.7	(1.7, 18.3)
20-35	15	1.75	8.8	(6.6, 23.4)
30-40	10	1.33	9.0	(1.0, 19.0)
**30-45**	**15**	**1.50**	**9.1**	**(5.8, 24.1)**

Lastly, we conservatively anticipate an SD of around 12 (DBP) to 15 (SBP) for the BP measurements resulting in excellent precision to estimate the mean BP post-intervention in each of the groups. Conservatively, assuming that 30% of the randomized sample ultimately have usable blood pressure measurements and a randomized sample size of 170 per arm, the precision (margin of error) of the two-sided 90% CI on mean DBP will be +/- 2.3 mmHg and for SBP will be +/- 3.5 mmHg. These calculations are for the individual arms. The difference in means for the two arms will be estimated to +/- 4.6 mmHg for DBP and to +/-6.9 mmHg for SBP. If a larger number of subjects have BP measurements the precision of these estimates will be improved.

<u>1.2 Data analysis</u>

The data, including the outcome data, baseline demographics and clinical characteristics, will be explored, overall and by arm, using graphical tools and summary statistics. The primary analysis will compare the proportion of patients participating in each study arm using a Z-test. We will calculate estimates and 90% confidence intervals (CI) for the rates of enrollment for each group and the difference in rates and the 90% CI on the difference. Using a logistic regression model, we will explore whether participation in an arm differs by baseline demographic and clinical characteristics

We will also compare the mean rates of participation in BP measurements in the two arms using a generalized estimating equations (GEE) model that includes the number of BP measurements submitted by each patient with a separate offset for the number of measurements requested. Because participation in the arms may differ by demographic/clinical characteristics, we will secondarily adjust the model for these variables. Similarly, using a logistic regression model, we will estimate the odds of engagement (defined as submitting at least 50% of the requested BP measurements over the duration of the program) between recruitment arms, again with adjustment for demographic and clinical characteristics. Similarly we will estimate the total proportion with controlled BP in the program arms and usual care arm. We will quantify the mean, standard deviation (SD), median and interquartile range of the BP measurements at baseline and at the conclusion of the study. Using a linear model, we will determine whether mean BP differs by arm after adjustment for baseline BP and use the model to provide estimates of both final BP as well as changes in blood pressure from baseline. Final blood pressure measurements for home monitoring at end of study will be the average of the final 3 patient submitted blood pressures for those enrolled in the program. We will use the blood pressure measurement(s) from the most recent in-person clinic visit for all arms.

Additional outcomes include an evaluation of the escalation criteria, escalation frequency, and text message content value.

Study Design

<u>1.1 Design</u>

This is randomized control trial of recruitment methodologies with approximately 425 patients being recruited to participate in a 6 month escalating, stepped remote monitoring program to control blood pressure. Eligible participants will be randomized in a 2:2:1 ratio to three recruitment arms in variable blocks of 5 and 10 using a computer-generated randomization algorithm: A) opt-in recruitment (mailed letter + recruitment phone call), B) opt-out recruitment (mailed blood pressure cuff and letter with opt-out messaging + recruitment phone call), or C) usual care (no contact). Using the Way to Health (WTH) platform for bidirectional text messaging with patients, we will iterate on the design of the program and implement learnings at 6-8 week intervals to identify best methods for participant engagement, messaging content, escalation criteria, escalation frequency, titration period and order of steps.

1.2 Consent Process

We are requesting a waiver of the requirement to document consent and HIPAA authorization with a signature for participants randomized into a recruitment arm for this study, since we believe the research presents no more than minimal risk of harm to subjects and involves no procedures for which written consent is normally required outside of the research context. All participants being enrolled in this study will be enrolled via a remote recruitment process, therefore we will read the IRB approved Consent/HIPAA script over the phone to each patient and ask them to provide verbal consent and HIPAA authorization for use of their data in the study. A copy of the consent form will also be mailed to the participant's home address for their review prior to receiving any recruitment calls from research staff. As all recruitment will be done over the phone, and as there are no in-person visits required as part of this study, a waiver of the requirement to document consent and HIPAA authorization with a signature is requested.

Participants who are randomized to the control arm will not be recruited for participation in the remote monitoring program. We request a waiver of consent for participants randomized to this study arm, as the consent process itself may influence the study outcomes, we are requesting a waiver of consent since we believe the research presents no more than minimal risk of harm to this group of participants.
The remote monitoring program has been designed in collaboration with Penn Family Care clinicians and the program is clinically appropriate for those with uncontrolled hypertension. All participants will continue to receive standard clinical care. All participants randomized to the recruitment arms will also be informed that this program is voluntary and that they can stop participating at any time.

Study Duration

We expect recruitment to take approximately 5 months. The program itself will be 6 months from the date the participant agrees to participate, and we will need an additional 3 months to complete qualitative interviews with a random sample of 50 participants, as well as clinicians from Penn Family Care. Including data analysis and manuscript preparation, total study duration should not exceed 15 months.

Resources Necessary for Human Research Protections

Dr. Mehta along with Project Manager (Catherine Reitz) and all Clinical Research Coordinators ~~(Lauren Ianotte, Kiernan McNelis, and Caitlin McDonald)~~ are adequately informed of the protocol and adequately qualified to conduct research via training required for medical doctors/students and research coordinators. All are up to date with HIPAA and CITI training.

Detailed Standard Operating Procedure documents for the project will be accessible to all members of the research team, which will keep research staff informed about the protocol and their related duties. There are adequate facilities to conduct the research.

Target Population
The targeted population is patients aged 18-75 within the Penn Family Care practice with at least ~~1~~ 2 in-person office visit within the prior ~~12~~ 24 months and the last blood pressure readings exceeding recommended HTN guidelines in that time, including the most recent.

Subjects Enrolled by Penn Researchers
425

Subjects Enrolled by Collaborating Researchers
0

Accrual
Participants in this study will be identified via electronic medical records review by our study staff. The study staff will work with UPHS to create a filter for the electronic medical record system that will generate a list of patients who may meet the study criteria. A study coordinator will review the list of patients to determine eligibility. If patients meet the minimal requirements they will be randomized and recruited according to study arm. Based on a previous pilot in the same clinical population, we estimate that 31% of eligible patients offered the remote monitoring program will agree to participate in the opt-in recruitment arm. We plan to enroll 425 patients in the study in a 2:2:1 ratio, with 170 in each recruitment arm, and 85 in the usual care/no contact arm. Considering a two-sided type I error rate of .05, we have 80% power to detect an increase in recruitment of 15%, with opt-out compared to opt-in enrollment. Recruitment will end once 425 eligible participants have been randomized.

Key Inclusion Criteria
1. Between 18 and 75 years old with HTN (ICD-10 code I10)
2. Has had at least ~~one~~ two office visits at Penn Family Care (PFC) within the past ~~12 months~~2 years (at time of chart review), with ~~the last visit having a~~at least 2 BP readings exceeding HTN guidelines (150/90 if ≥60 or, 140/90 if ages 21-59 yrs or has CKD or diabetes), including the measurement from the most recent visit..
3. Must have a cellular phone with texting capabilities
4. Must be prescribed at least one medication for hypertension

Key Exclusion Criteria
1. Has metastatic (Stage IV) cancer
2. Has end stage renal disease
3. Has congestive heart failure
4. Has dementia
5. BMI >= 50
6. Is Non-English speaking requiring a translator

Vulnerable Populations
No vulnerable populations are specifically included or excluded in the research study. We will include all eligible patients in the Penn Family Care practice.

Populations Vulnerable to Undue Influence or Coercion
We are not specifically targeting any vulnerable populations.

Subject Recruitment
Participants in this study will be identified via electronic medical records review by our study staff. The

study staff will work with UPHS to create a filter for the electronic medical record system that will generate a list of patients who may meet the study criteria. A study coordinator will review the list of patients to determine eligibility. If patients meet the minimal requirements they will be randomized to three recruitment arms in variable blocks of 5 and 10 using a computer-generated algorithm, and recruited according to study arm. Recruitment will end once 425 eligible participants have been randomized and all contact attempts exhausted.

Up to three potential support partners will be identified by the participant at the time of their enrollment. If the participant reports at least 3 of 10 elevated blood pressure measurements (as defined by guidelines), the CRC will make up to 3 attempts to contact and enroll the first support partner provided. If they decline or are not reachable, the CRC will make up to 3 call attempts each with the second and third support partners until a partner has been enrolled or until all call attempts have been exhausted with all partners provided. Support partners will be informed of the research the participant is part of, and offer their verbal agreement to participate in the program.

Subject Compensation
Participants selected for interview will be offered a $50 Greenphire virtual ClinCard for their time completing the interview. ~~will not be compensated for their participation.~~

Procedures
Recruitment: A data pull from Clarity will be used to identify potentially eligible patients at the Penn Family Care family medicine practice who have had at least 2 office visits in the last 2 years. Eligible patients will have a diagnosis of HTN, be taking at least one BP medication, have a valid cell phone and text messaging plan, and have ~~an~~ at least 2 elevated BP measurements per guidelines within the last 2 years, including at the most recent visit. Patients will be excluded if they have a history of CHF, dementia, BMI >=50, ESRD or metastatic cancer. In variable blocks of 5 and 10 using a computer-generated algorithm, eligible patients will be randomized into three recruitment groups: A) opt-in recruitment (mailed letter + recruitment phone call), B) opt-out recruitment (mailed blood pressure cuff + letter with opt-out messaging + recruitment phone call), or C) usual care (no contact).

Patients in the usual care arm will not be contacted by study staff, they will not receive a blood pressure cuff, and they will not receive any text messaging or any component of the stepped program.

Patients in the opt-in recruitment arm will receive a letter describing the program and inviting them to participate. Along with the letter they will receive an informational brochure and a copy of the informed consent form. Patients in this arm will then receive a recruitment phone call explaining the program and consenting the participant verbally. If the participant consents to participate in the remote monitoring program, to receive text messaging, and to connection to care, the research coordinator will mail a blood pressure cuff and begin the remote monitoring program for the patient in the Way to Health platform. Patients will also be asked to provide the names of up to 3 friends or family members to serve as a potential support partner and to confirm correct BP cuff sizing.

Patients assigned to opt-out recruitment will receive a blood pressure cuff with instructions in the mail along with a letter describing the program and opt-out framing regarding their participation, as well as a brochure and a copy of the informed consent form. Patients in this arm will receive a recruitment phone call similar to recruitment arm A, explaining the program and consenting the participant verbally. If the participant consents to participate in the remote monitoring program, to receive text messaging, and to

connection to care, the research coordinator will begin the remote monitoring program for the patient in the Way to Health platform. Patients will also be asked to provide the names of up to 3 potential support partners and to confirm correct BP cuff sizing.

For both the Opt-In and Opt-Out recruitment arms, the Research Coordinators will make up to 3 phone call attempts to reach the patient. If the patient cannot be contacted after 3 attempts, they will be marked as unreachable and no further attempts to recruit the patient will be made.

Participants in both recruitment groups will proceed through the same remote monitoring program regardless of recruitment method and in collaboration with their care team at the Penn Family Care practice. Participants of either group may end their participation in the program at any time. Participants of either recruitment group who are found to be unreachable will be mailed a debrief letter explaining their participation in the program.

Remote Monitoring Program: All patients will begin the program in the "Enhanced Baseline" phase and will escalate through the 5 program steps only as needed for BP control. All patient submitted BP measurements will be visible to clinicians from the WTH integrated BP flowsheet. Each step of the intervention and escalation criteria are described below (also see attached flowsheet).

Step 1 - Enhanced Baseline: patients will receive BP prompts three times each week ("What is your blood pressure today?"), a medication adherence prompts~~ ("Have you taken your [drug name] as prescribed this week?")~~, positive feedback on BP measurements if controlled ("You've submitted three controlled measurements in a row! Great job!"), lifestyle messaging (diet, exercise, self-efficacy, and other tips relevant for controlling hypertension), and feedback and notice of escalation to the nursing pool ("Hi there PARTICIPANT_FIRSTNAME. It looks like you're struggling to keep your BP controlled, some of your measurements have been a little high lately. We've notified your Care Team and will step up your intervention. You may hear from your care team by text message (from #215-650-8055) in the next few days ❤ ~~You've submitted three elevated measurements in a row, we will notify your Care Team and step up your intervention~~"). If the patient submits any 3 elevated readings (per guidelines) ~~in a 3-week period~~out of the last 10 measurements, we will notify the nursing pool consisting of at least one designated nurse (RN) and one Nurse Practitioner (NP), and step the patient to the next level of the program, otherwise they will remain in this step through the end of their 6-month period.

Severely elevated readings (≥180/110) will receive an automated response informing them that the reading is high and request the measurement be taken again and resubmitted after 15 minutes. Patients will further be informed to contact the practice if it remains elevated. After the second reading is obtained, both measurements will be routed to the ~~nursing pool~~nurse practitioner for evaluation. If no response is re-submitted after 15 minutes, a prompt will be sent to the patient to resubmit, along with instructions to contact the practice if they are experiencing symptoms; the initial measurement will be routed alone. Extremely low measurements (<90/50) will be handled in the same way.

Step 2 - Enhanced Cheerleading: If the participant reports at least 3 of 10 elevated blood pressure measurements (as defined by guidelines), the CRC will make up to 3 attempts to contact and enroll the first support partner provided during the recruitment phone call. If the first support partner declines or is not reachable, the CRC will make up to 3 call attempts each with the second and third support partners until a partner has been enrolled or until all call attempts have been exhausted with all partners provided. Patients will continue to receive all components of the baseline program and the

patient will be notified that their support partner has been added. The support partner will receive BP measurement and adherence information submitted by the patient and encouraged to engage with the patient in personal conversation about his/her health ("[Patient Name] is struggling to keep their BP controlled, talk to them about how important their health is to you"). Positive feedback and milestones achieved by the patient will also be shared ("[Patient name has submitted 3 controlled BP measurements in a row, we'll let them know how proud you are unless you reply "N" to this message in the next 2 hours"), and messages will be personalized with names and relationships to the extent possible. If the patient submits 3 elevated readings (per guidelines) ~~in a 3-week period~~, out of 10 measurements we will notify the nursing pool and step the patient to the next level of the program, otherwise they will remain in this step through the end of their 6-month period.

Step 3 - Clinical Two-Way Texting: patients will continue to receive all components of the baseline program and enhanced cheerleading, and will additionally receive asynchronous text communication with the nursing pool for coaching and clarity related to medication adherence, symptoms, and/or any lifestyle factors that may need adjustment prior to medication titration. The nursing pool will be able to access the SMS inbox for each patient directly from the patient record. If the patient 3 elevated readings (per guidelines) ~~in a 3-week period~~ out of 10 measurements, we will notify the nursing pool and step the patient to the next level of the program, otherwise they will remain in this step through the end of their 6-month period. Asynchronous texting with the nursing pool may not continue if coaching is successful and BP is successfully controlled at this step, but will remain an option for the clinical team through the remainder of the patient's program.

Step 4 - Titration: patients will continue to receive all components of the three previous steps. The designated Nurse Practitioner will review patient submitted BP measurements through the integrated flowsheet, results at each step of the program and any information gathered through Clinical 2-Way Texting to make dosing or medication changes or add additional medications for BP control. Once the medication change has been implemented, adoption and adherence will be confirmed through Clinical 2-Way texting. If after a 4-week adoption period the patient again submits at least 3 elevated readings (per guidelines) ~~in a 3-week period~~ out of 10 measurements, we will notify the nursing pool and step the patient to the next level of the program, otherwise they will remain in this step through the end of their 6-month period.

Step 5 - Office/Telemedicine Visit: patients will continue to receive all components of the four previous steps. The Nurse Practitioner will meet with the patient in the office or via a virtual/telemedicine visit for additional clinical workup, referral, or additional medication changes. The patient will have this visit and remain in this phase through the end of their 6-month period. If the patient 3 elevated readings (per guidelines) ~~in a 3-week period~~ out of 10 measurements, we will notify nursing pool and they will, at their discretion, implement additional medication changes, see the patient again, or refer for additional clinical workup elsewhere.

The nurse practitioner or nursing pool will be notified of all escalations. At any point of escalation to the next step, the RN or NP may choose to directly escalate to step 4 or Step 5 for titration or a visit at his or her discretion, skipping any steps in between as deemed appropriate.

Engagement: If a participant does not engage with the intervention via text message (and does not notify the study team of an absence) for a minimum of 7 events (approximately 2 weeks), the participant will receive a text message prompt to try to re-engage them with the program. If after an additional 7 events pass without engagement, the RC will reach out to the participant by phone to assess the issue and make

another attempt to re-engage with the program. Finally, if additional 7 events (a total of approximately 6 weeks) pass without engagement, the RC will mail a letter to the patient's home. The patient will only be unenrolled from the program if they explicitly request it or opt-out of text messaging.

Assessment/Iteration: In 6-8 week intervals, we plan to assess the number of patients who are actively engaged with the program, the number of patients at each step, the percent controlled at each step, and the number of patients skipped directly to Step 4 or Step 5 by a member of the Nursing Pool. We will also evaluate participant engagement, escalation criteria, escalation frequency, and text message content value and adjust as needed to improve BP control among our participants.

Qualitative Assessment: Participants who agree to participate in the remote monitoring program will be assigned to participation groups based on the number and timing of blood pressures submitted to the program, as well as on their engagement with the clinician performing patient follow-up if blood pressures were escalated for not being controlled. Within each of these participation groups, participants will be randomly selected for an interview to collect qualitative data regarding their level of participation, barriers or supports to participation, perceptions about the program and stepped levels of care, and opportunities for improvement in the remote monitoring program. Rather than pre-specify a number of patients that must be interviewed per group, the research team will be attentive to thematic saturation and use this as a guide that a sufficient number of interviews has been reached. ~~We will interview a random subset of 50 patients to assess final patient engagement and gather additional qualitative insights about their experience and perception with the program.~~ We anticipate these interviews to take no more than ~~15-20~~20-30 minutes by phone. Research staff will make no more than three attempts to speak directly with the subject. Participants will be offered a $50 virtual Greenphire ClinCard for their time completing the interview, and will be informed the interviews will be recorded and transcribed and must agree to participate in the interview before it begins. Their agreement will be recorded in REDCap. Similarly, we will interview the clinicians participating in the Nursing Pool and a random set of PCPs whose patients participated in the program, to assess their experience and perception of the program.

Analysis Plan

Irrespective of whether the differences between arms achieve statistical significance, the study will provide valuable information about recruitment rates that can be used to guide study design, and particularly recruitment goals, for future clinical trials. Table 1 shows that if the observed recruitment rate for the study is 30% we can with high confidence assume that the actual rate in the overall population lies between 24 and 36%. In contrast if the observed rated is 45%, we can, with high confidence, assume that the actual rate in the overall population is considerably higher, between 39 and 51%. This study can also provide estimates of differences in the enrollment rates between recruitment methods. For example, Table 2 shows that the observed rate difference is 15% (for individual group rates of 30-45%), then the 90% CI suggests that the actual rates in the population lie between 6-24%. This information could prove valuable in assessing for example cost effectiveness of the proposed opt-out strategy.

Table 1: Estimation of enrollment rates for individual recruitment strategies for two-sided 90% CI (n=170 per group)		
Observed Rate (%)	½ width of 90% CI (Margin of error %)	Endpoints of 90% CI (%)
10	3.8	(6.8, 14.4)
20	5.0	(15.4,25.5)

**30**	**5.7**	**(24.6, 36.1)**
40	6.1	(34.0, 46.3)
**45**	**6.2**	**(38.9 51.3)**

Table2: Estimation of enrollment differences with two-sided 90% CI (170 per group)				
Observed Rates (%)	Rate difference (%)	Rate Ratio	½ width of 90% CI (Margin of error)	Endpoints of 90% CI (%)
10-20	10	2.0	6.9	(3.1, 16.9)
10-25	15	2.5	7.2	(7.8, 22.2)
20-30	10	1.5	7.7	(1.7, 18.3)
20-35	15	1.75	8.8	(6.6, 23.4)
30-40	10	1.33	9.0	(1.0, 19.0)
**30-45**	**15**	**1.50**	**9.1**	**(5.8, 24.1)**

Lastly, we conservatively anticipate an SD of around 12 (DBP) to 15 (SBP) for the BP measurements resulting in excellent precision to estimate the mean BP post-intervention in each of the groups. Conservatively, assuming that 30% of the randomized sample ultimately have usable blood pressure measurements and a randomized sample size of 170 per arm, the precision (margin of error) of the two-sided 90% CI on mean DBP will be +/- 2.3 mmHg and for SBP will be +/- 3.5 mmHg. These calculations are for the individual arms. The difference in means for the two arms will be estimated to +/- 4.6 mmHg for DBP and to +/-6.9 mmHg for SBP. If a larger number of subjects have BP measurements the precision of these estimates will be improved.

<u>1.2 Data analysis</u>

The data, including the outcome data, baseline demographics and clinical characteristics, will be explored, overall and by arm, using graphical tools and summary statistics. The primary analysis will compare the proportion of patients participating in each study arm using a Z-test. We will calculate estimates and 90% confidence intervals (CI) for the rates of enrollment for each group and the difference in rates and the 90% CI on the difference. Using a logistic regression model, we will explore whether participation in an arm differs by baseline demographic and clinical characteristics

We will also compare the mean rates of participation in BP measurements in the two arms using a generalized estimating equations (GEE) model that includes the number of BP measurements submitted by each patient with a separate offset for the number of measurements requested. Because participation in the arms may differ by demographic/clinical characteristics, we will secondarily adjust the model for these variables. Similarly, using a logistic regression model, we will estimate the odds of engagement (defined as submitting at least 50% of the requested BP measurements over the duration of the program) between recruitment arms, again with adjustment for demographic and clinical characteristics. Similarly we will estimate the total proportion with controlled BP in the program arms and usual care arm. We will quantify the mean, standard deviation (SD), median and interquartile range of the BP measurements at baseline and at the conclusion of the study. Using a linear model, we will determine whether mean BP differs by arm after adjustment for baseline BP and use the model to provide estimates of both final BP as well as changes in blood pressure from baseline. Final blood pressure measurements for home monitoring at end of study will be the average of the final 3 patient submitted blood pressures for those enrolled in

the program. We will use the blood pressure measurement(s) from the most recent in-person clinic visit for all arms.

Additional outcomes include an evaluation of the escalation criteria, escalation frequency, and text message content value.

Data Confidentiality

Paper-based records will be kept in a secure location and only be accessible to personnel involved in the study. Computer-based files will only be made available to personnel involved in the study through the use of access privileges and passwords. Prior to access to any study-related information, personnel will be required to sign statements agreeing to protect the security and confidentiality of identifiable information. Wherever feasible, identifiers will be removed from study-related information. Precautions are in place to ensure the data is secure by using passwords and encryption, because the research involves web-based surveys. Audio and/or video recordings will be transcribed and then destroyed to eliminate audible identification of subjects.

Subject Confidentiality

Information about study subjects will be kept confidential and managed according to the requirements of the Health Insurance Portability and Accountability Act of 1996 (HIPAA). All PHI will be maintained on UPHS servers. Source documents are maintained in PennChart. No source documents will be printed or maintained in paper form at the study site. Data from PennChart will be recorded in Penn Medicine's REDCap system. The investigator and study team (which includes the project manager and research coordinators) will have access to PHI within PennChart and REDCap. We will label all PHI within REDCap as identifiable information so that de-identified exports are possible. All reports that include identifiable information will be stored on the Innovation Center secure drive, maintained behind the UPHS firewall. Once data analysis and manuscripts have been published, the databases will be removed from REDCap and the data will be de-identified on the secure drive. This de- identified dataset will be stored for up to five years after analysis is complete and manuscripts have been published. Once analysis is completed and any manuscripts are published, we will retain PHI no longer than seven years in accordance with government regulations, applicable policies, and institutional requirements. Phone calls will be transcribed using Datagain Transcription, a HIPAA compliant transcription service. Datagain's system controls, database architecture and internal policies provide HIPAA compliance.

Database Security/Protection Against Risk

Information about study subjects will be kept confidential and managed according to the requirements of the Health Insurance Portability and Accountability Act of 1996 (HIPAA). All PHI will be maintained on UPHS servers. Source documents are maintained in PennChart. No source documents will be printed or maintained in paper form at the study site. To assure that patient, physician and other informant confidentiality is preserved, individual identifiers (such as name and medical record number/physician billing identifier) are stored in a single password protected system that is accessible only to study research, analysis and IT staff. This system is hosted on site at The University of Pennsylvania (UPenn) and is protected by a secure firewall. Once a participant is in this system, they will be given a unique study identification number (ID). Any datasets and computer files that leave the firewall will be stripped of all identifiers and individuals will be referred to by their study ID. The study ID will also be used on all analytical files.

The initial patient information collected for screening and recruitment will consist of name, address, phone number, demographic information such as race and income, and medication adherence practices. As our patient population is patients in the UPHS health system, the majority of this information will come from Electronic Chart reviews.

Sensitive Research Information

This research does not involve collection of sensitive information about the subjects that should be excluded from the electronic medical record.

Subject Privacy

We will only interact with the subsample of subjects with which we plan to enroll in the study. With these participants, we will conduct phone calls in a private area. When we call subjects, we will confirm the identify before beginning the recruitment process.

Data Disclosure

Blood pressure measurements will be disclosed to the designated centralized nursing pool within Penn Family Care, and may be provided to the primary care physician as deemed appropriate by the Nurse Practitioner, or if they reach or surpass a pre-established threshold determined to be an immediate danger to the subject's health. Greenphire ClinCard and the Office of Finance at the University of Pennsylvania will receive participant names, address, and date of birth for subject compensation payments.

Protected Health Information/Data Protection

- Name
- Street address, city, county, precinct, zip code, and equivalent geocodes
- All elements of dates (except year) for dates directly related to an individual and all ages over 89
- Telephone and fax numbers
- Medical record numbers
- Biometric identifiers, including finger and voice prints

Consent Process

1.1 Overview

We are requesting a waiver of the requirement to document consent and HIPAA authorization with a signature for participants randomized into a recruitment arm for this study, since we believe the reseach presents no more than minimal risk of harm to subjects and involves no procedures for which written consent is normally required outside of the research context. The remote monitoring program has been designed in collaboration with Penn Family Care clinicians and the program is clinically appropriate for those with uncontrolled hypertension. All participants will continue to receive standard clinical care. All participants being enrolled in this study will be enrolled via a remote recruitment process, therefore we will read the IRB approved Consent/HIPAA script over the phone to each patient and ask them to provide verbal consent and HIPAA authorization for use of their data in the study. A copy of the consent form will also be mailed to the participant’s home address for their review prior to receiving any recruitment calls from research staff. All participants randomized to the recruitment arms will also be informed that this program is voluntary and that they can stop participating at any time. For these reasons, and because all

recruitment will be done remotely and no in-person visits required as part of this study, a waiver of the requirement to document consent and HIPAA authorization with a signature is requested.

Participants who are randomized to the control arm will not be recruited for participation in the remote monitoring program. We request a waiver of consent for participants randomized to this study arm, as the consent process itself may influence study outcomes, because we believe the research presents no more than minimal risk of harm to participants.

Verbal consent will be obtained from the subsample with whom we plan to conduct post-intervention interviews. These subjects will be informed about the purpose of the phone call, asked if they would like to participate and if the phone call can be recorded. Since the interviews will be conducted by phone, verbal consent will be obtained and recorded in REDCap.

<u>1.2 Children and Adolescents</u>
Not applicable

<u>1.3 Adult Subjects Not Competent to Give Consent</u>
Waiver of consent is being requested.

Waiver of Consent
<u>1.1 Minimal Risk</u>
The research presents no more than minimal risk of harm to subjects and involves no procedures for which written consent is normally required outside of the research context. The research related activity is the randomization of subjects to different types of recruitment strategies.

<u>1.2 Impact on Subjects Rights and Welfare</u>
Participation in the remote monitoring program is completely voluntary and the participants rights and welfare will not be adversely affected by the waiver of documentation of consent.

<u>1.3 Waiver Essential to Research</u>
As the consent process itself may influence study outcomes we are requesting a waiver of consent for participants randomized to the control arm because we believe the research presents no more than minimal risk of harm to participants, and explaining the remote monitoring program to a participant in the Usual Care arm may influence the patient's behavior during the program period. We are requesting a waiver of the requirement to document consent and authorization with signature for participants enrolled in the intervention because we believe the research presents no more than minimal risk of harm to subjects and involves no procedures for which written consent is normally required outside of the research context.

<u>1.4 Written Statement of Research</u>
Upon completion of the study, we will send a letter to patients randomized to the remote monitoring arms that we are unable to reach for recruitment, explaining that we ran a quality improvement trial focused on enrollment and remote monitoring of blood pressure.

Potential Study Risks

As this study does not involve any medical decision making and only tests the use of behavioral approaches to encouraging patients to participate in a remote monitoring program for hypertension management, we consider this study minimal risk. The primary risk would be from a breach of confidentiality involving medical records reviews and monitoring of hypertension medication and blood pressure adherence with text messaging which will be maintained on the Way to Health platform. This risk has been mitigated by extensive privacy protection protocols, a highly secure data storage system, and a plan to remove identifiers from the data wherever possible. In addition, all personnel will be held to high standards of upholding confidentiality and safeguarding patient privacy.

Potential Study Benefits

The immediate benefits of this study for participants may include an improvement in adherence to behaviors and medications that have been proven to be effective in improving patient outcomes. It is possible that the benefits for many participants will be minimal. However, as mentioned, we believe the risks are also minimal. Knowledge gained from the study will assist in development of recruitment methods for remote monitoring interventions in other high risk patient populations in which non-adherence rates are high and blood pressure remains uncontrolled. The potential public health impact of a successful recruitment to an intervention to improve BP control is great and could reduce the number of deaths from health related outcomes in the United States each year.

Data and Safety Monitoring

The study PI will be responsible for monitoring the data and safety of the study as well as the program, and ensuring the ongoing quality and integrity of the research study. The investigator will permit study-related monitoring, audits and inspections by government regulatory authorities and University compliance officers.

Risk/Benefit Assessment

This study is designed to test recruitment strategies for enrollment in a remote monitoring program that incorporates many components of prior interventions that have previously demonstrated promise in feasibility, clinical workflow, and potentially relevant improvement in BP. We believe the combination of these approaches in this intervention will provide the research and public health communities with important information that can lead to broad generalizability in treating people at risk for the above mentioned chronic diseases and death nationally, as these types of programs could be set up by healthcare systems to be broadly utilized. However, successful recruitment and engagement of patients is critical to the success of the program itself, particularly in a remote management context. With minimal risks, the potential public health impact of a successful recruitment to a program to improve BP control is great and could reduce the number of deaths from health related outcomes in the United States each year.

Summary of Protocol Changes Modifications LOG

Protocol: SupportBP 2.0
University of Pennsylvania Principal Investigator: Shivan Mehta, MD

Date of Submission	Description of Modification	Rationale for Modification	Approval date
12/7/2020	Initial Submission		12/23/2020
1/11/2021	Modification<br>a. Inclusion criteria<br>b. Texting content/messaging<br>c. Clinical notifications<br>d. Patient engagement outreach	a. Updated from 1 visit in past 1 year with elevated BP per guidelines to 2 visits in 2 years with elevated BP, including at most recent visit<br>b. Minor content updates to language, no substantive changes<br>c. Updates to escalation details and process<br>d. Added for patients disengaged for 2 weeks	1/14/2020
6/14/2021	Modification<br>a. Add research coordinator	a. Additional staff needed for recruitment	6/27/2021
8/4/2021	Deviation<br>a. Logic processing error in automation led to 13 missed escalations between 3/31/21 and 7/7/21		8/5/2021
11/15/21	Continuing Review Submission		11/16/2021
6/10/2022	Modification<br>a. Updated follow-up patient interview plan<br>b. Add interview team, remove research coordinators	a. Collaborated with a group with interview expertise to get more informative and complete qualitative data<br>b. Added interview staff, removed CRCs (intervention complete)	6/28/2022
11/2022, 10/2023, 9/2024	Continuing Review Submissions		11/16/2022, 10/19/2023
9/9/2024	Modification<br>a. Remove Staff	a. Removed interview staff and staff no longer working on project	9/23/2024

Appendix A: Final Recruitment Materials

A1. Opt-in Brochure

Frequently Asked Questions

Am I eligible?

You're eligible for this program if you have hypertension and:

- ✓ You're between the ages of 18 and 75
- ✓ You're a patient at Penn Family Care
- ✓ You've had two blood pressure readings >150/90 (or >140/90 if aged 21-59)
- ✓ You have a cell phone with text messaging
- ✓ You're currently taking blood pressure medication

Is there any cost for the program?

There is no cost to you for participating in this program.

How did you get my information?

This program is operated by Penn Medicine and is for eligible Penn Family Care patients. You were chosen because this program may help you get better care.

How do I sign up?

Call a SupportBP team member today to find out more and enroll! You can reach us at [redacted] or email [redacted]pennmedicine.upenn.edu with the best time to reach you.

What's Next?

In a few days, a program coordinator will call to discuss the program. Please continue taking your meds as usual, the regular care you receive from your health care providers will not change.

When the program coordinator calls, they will review your eligibility, answer any questions you may have, and ask for your participation.

If you agree to participate, we will send you a blood pressure cuff to get started with the program.

The program coordinator will explain what to expect in more detail.

You may also contact us directly by calling [redacted] or by emailing us at [redacted]pennmedicine.upenn.edu with questions or to enroll at any time.

SupportBP

SupportBP

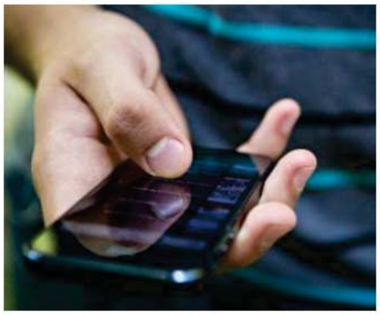

Penn Medicine

Department of Family Medicine & Community Health

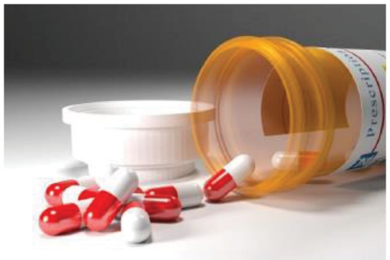

About SupportBP

The SupportBP program is designed to help people improve their health and better manage their blood pressure.

You are being invited to participate in this program in the hopes that your involvement can improve not only your own care, but also the care we provide to patients in the future.

The SupportBP program is working with Penn Family Care and Penn Medicine at the University of Pennsylvania. Penn Medicine is dedicated to high-quality patient care and service – and to advancing medical science through research and innovation in care delivery.

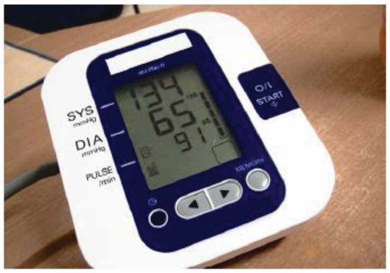

Program Benefits

Remote Blood Pressure Monitoring

You will receive a home blood pressure cuff and regular text-based reminders to take and report your home blood pressure readings. You'll also be asked to report your medication adherence, receive messaging related to lifestyle management of hypertension, and receive weekly feedback from the program.

Connection to your Care Team

The measurements you text us will be visible to your physician. If you report multiple measurements that are elevated, we will flag these for your physician to review.

Individualized Program

We know that everyone needs different levels of support. If you report multiple elevated BP measurements, we will add the next level of support to your program, which may include additional accountability from a friend or family member, a change or increase in medication, or a visit with your physician until, we are able to get your blood pressure within appropriate limits.

Improving Patient Care

We support patients as they develop healthy habits, such as taking medication every day and regularly monitoring blood pressure to better control blood pressure. Your participation will not only be helping you, but also many others!

What Else Will I Need to Do?

Participation in the program will last 6 months. All you will need to do is regularly monitor your blood pressure, report your BP measurements, take your medications as prescribed, and receive and reply to our text messages.

Your primary care physician will be notified about your participation and will also be notified about blood pressure measurements you report.

The regular care you receive from your health care providers will not change.

That's it!

Questions?

[redacted]
[redacted]pennmedicine.upenn.edu

Penn Medicine

Department of
Family Medicine &
Community Health

A2. Opt-Out Brochure

Frequently Asked Questions

Am I eligible?

You're eligible for this program if you have hypertension and:

- ✓ You're between the ages of 18 and 75
- ✓ You're a patient at Penn Family Care
- ✓ You've had two blood pressure readings >150/90 (or >140/90 if aged 21-59)
- ✓ You have a cell phone with text messaging
- ✓ You're currently taking blood pressure medication

Is there any cost for the program?

There is no cost to you for participating.

How did you get my information?

This program is operated by Penn Medicine and is for eligible Penn Family Care patients. You were chosen because this program may help you get better care.

How do I sign up?

You're already in! A SupportBP team member will call to confirm your participation in a few days. Once confirmed, your program will begin. To confirm sooner, call [redacted] or email [redacted]ennmedicine.upenn.edu with the best time to reach you.

What's Next?

In a few days, a program coordinator will call to discuss the program. Please continue taking your meds as usual, the regular care you receive from your health care providers will not change.

When the program coordinator calls, they will review your eligibility, answer any questions you may have, and confirm your participation.

You may keep the blood pressure monitor included in this package even if you do not confirm your participation. Once confirmed, you will be started in the program. The program coordinator will explain what to expect in more detail.

You may also contact us directly by calling [redacted] or by emailing us at [redacted]ennmedicine.upenn.edu with questions or to discuss your participation at any time.

SupportBP

SupportBP

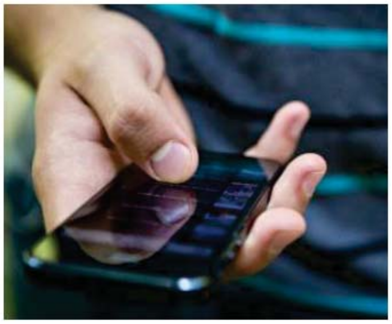

Penn Medicine

Department of Family Medicine & Community Health

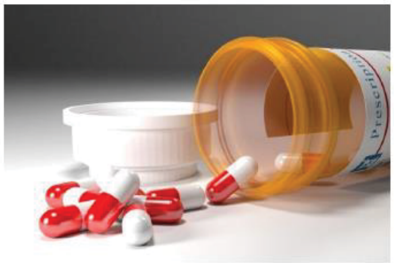

About SupportBP

The SupportBP program is designed to help people improve their health and better manage their blood pressure.

You are being invited to participate in this program in the hopes that your involvement can improve not only your own care, but also the care we provide to patients in the future.

The SupportBP program is working with Penn Family Care and Penn Medicine at the University of Pennsylvania. Penn Medicine is dedicated to high-quality patient care and service – and to advancing medical science through research and innovation in care delivery.

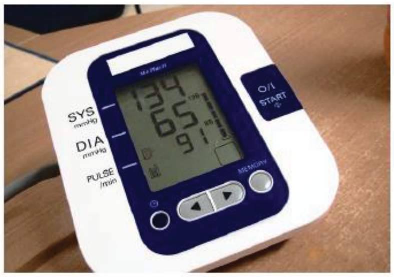

Program Benefits

Remote Blood Pressure Monitoring

You will receive regular text-based reminders to take and report your home blood pressure readings. You'll also be asked to report your medication adherence, receive messaging related to lifestyle management of hypertension, and receive weekly feedback from the program.

Connection to your Care Team

The measurements you text us will be visible to your physician. If you report multiple measurements that are elevated, we will flag these for your physician to review.

Individualized Program

We know that everyone needs different levels of support. If you continue to report multiple elevated BP measurements, we will add the next level of support to your program, which may include additional accountability from a friend or family member, a change or increase in medication or a visit with your physician until we are able to get your blood pressure within appropriate limits.

Improving Patient Care

We support patients as they develop healthy habits, such as taking medication every day and regularly monitoring blood pressure to better control blood pressure. Your participation will not only be helping you, but also many others!

What Else Will I Need to Do?

Participation in the program will last 6 months. All you will need to do is regularly monitor your blood pressure, report your BP measurements, take your medications as prescribed, and receive and reply to our text messages.

Your primary care physician will be notified about your participation and will also be notified about blood pressure measurements you report.

The regular care you receive from your health care providers will not change.

That's it!

Questions?

[redacted]
[redacted]@pennmedicine.upenn.edu

Penn Medicine

Department of Family Medicine & Community Health

Appendix B: Final Messaging Content

SUPPORT BP 2.0 TEXT MESSAGING CONTENT

PATIENT MESSAGING

INTERVENTION OPT-IN (ARM 1)

Potential participant's information will be entered into W2H and randomized. Potential participants randomized to Arm 1 will be sent an informational letter & brochure about SupportBP2.0 asking them to call to enroll. Participants who do not respond to the letter will be called up to three times in efforts to enroll them. During the enrollment process, participants preferred cell phone number and the cell phone numbers for up to three support partners will be collected, participants will be mailed a blood pressure cuff and the SupportBP program will begin 6 days later (to allow for the BP cuff to arrive). The program itself will be identical for both arms with the exception of the initial welcome message and the timing of participant start.

INTERVENTION OPT-OUT (ARM 2)

Participant's information will be entered into W2h and randomized. Potential participants randomized into Arm 2 will be sent an introductory letter to the program (with an opt-out option), a brochure and a blood pressure monitoring cuff. Participants will be asked to call the study team to complete the enrollment process. If patients do not reach out to study staff, they will be called up to three times to complete enrollment. During the enrollment completion process, participants preferred cell phone number and the cell phone numbers for up to three support partners will be collected, and the SupportBP program will begin immediately. The program itself will be identical for both arms with the exception of the initial welcome message and the timing of participant start.

CONTROL ARM (ARM 3)

Participants randomized into Arm 3 are never contacted and will not receive the SupportBP program. Their pre- and post-study blood pressure readings and other secondary outcome measures will be obtained from the EHR.

WELCOME MESSAGING

OPT-IN ARM (ARM 1)

Arm 1 Participants Only
Begins: Day -6, upon enrollment
Repeats: does not repeat

"Welcome to SupportBP, PARTICIPANT_FIRSTNAME! You'll receive your welcome packet, blood pressure cuff & instructions within about a week, and your first monitoring texts will start shortly after that. Questions? Call xxx-xxx-xxxx or email SupportBP@uphs.upenn.edu. Privacy: Please be advised that standard SMS texting is not secure, so other people may be able to see the information in these messages. Text "Bye" at any time to stop receiving these messages."

Arm 1 Participants Only
Begins: Day 0 (6 days after enrollment)
Repeats: Does not repeat

PARTICIPANT_FIRSTNAME, your SupportBP program starts today! You can expect to receive a text to measure and submit your blood pressure (BP) three times each week, and a text about how you're taking your medications once a week. We will share your BP measurements with your care team, and add additional support to your program if you need it, as we go. We

hope you find this program helpful! Call us if you have any questions: xxx-xxx-xxxx. After reading through the BP monitoring instructions we sent you, do you feel confident in your ability to take your blood pressure properly? Please reply with either Y or N.

If Y: Great then you're ready to get started. If you ever decide you do need help, please text the word SUPPORT at any time to start a BP monitoring tutorial

If N: [begin BP MONITORING TUTORIAL]

If No Response: We didn't hear from you, but if you ever decide you do need help with how to monitor your blood pressure, please text the word SUPPORT at any time to start a BP monitoring tutorial.

OPT-OUT ARM (ARM 2)

Arm 2 Participants Only
Begins: Day 0, Upon enrollment
Repeats: does not repeat

Welcome to SupportBP, PARTICIPANT_FIRSTNAME, and thank you for confirming your participation! Please be advised that standard SMS texting is not secure, so other people may be able to see the information in these messages. Text "Bye" at any time to stop receiving these messages."

PARTICIPANT_FIRSTNAME, your SupportBP program starts today! You can expect to receive a text to measure and submit your blood pressure (BP) three times each week, and a text about how you're taking your medications once a week. We will share your BP measurements with your care team, and add additional support to your program if you need it, as we go. We hope you find this program helpful! Call us if you have any questions: xxx-xxx-xxxx. After reading through the BP monitoring instructions we sent you, do you feel confident in your ability to take your blood pressure properly? Please reply with either Y or N.

If Y: Great then you're ready to get started. If you ever decide you do need help, please text the word SUPPORT at any time to start a BP monitoring tutorial

If N: [begin BP Monitoring Tutorial]

If No Response: We didn't hear from you, but if you ever decide you do need help with how to monitor your blood pressure, please text the word SUPPORT at any time to start a BP monitoring tutorial.

BLOOD PRESSURE

AUTOMATED BP MEASUREMENT REMINDERS

Arms: 1 & 2
Begin: First Monday, Wednesday, or Friday after enrollment completed and blood pressure cuff received
Repeats on days: Monday, Wednesday, Friday
At: 8am

- Participant will receive one of "
  - "Good morning, <PARTICIPANT>. It's important to record your blood pressure every day."

  <u>OR</u>

- Taking your blood pressure regularly helps you manage it."

OR

- "Every day is another opportunity to take control of your health.

AND

→ What is your blood pressure today? (Ex. 120/80). Please respond within 24 hours."

ALL BP RESPONSES WILL FOLLOW THIS LOGIC

- **If valid input <180/110 and >=90/50 received, participant will receive one of:**
    - "{systolic_value}/{diastolic_value} has been recorded. Thank you." OR
    - Thanks for submitting your BP today, {systolic_value}/{diastolic_value} has been recorded 🙌 OR
    - We received your BP measurement, {systolic_value}/{diastolic_value}. Thanks for submitting!

- **If valid input ≥180 OR 110 received *[SEVERELY ELEVATED BP CONVERSATION]***
- "Your blood pressure ({systolic_value}/{diastolic_value}) is elevated. Please wait 15 minutes, check it again, and let us know what your new BP is (Ex. 120/80)"
    - **Recheck: If valid input <180/110 received**
        - "{systolic/diastolic} has been recorded. It's good that your measurement has come down, please continue to monitor closely. If the top measurement is consistently higher than 180 or the bottom measurement is consistently higher than 110, please call the office to speak to a clinician about your blood pressure: xxx-xxx-xxxx. We will also let your care team know."
    - **Recheck: If valid input ≥180/110 received**
        - ➔ "Your blood pressure ({systolic_value}/{diastolic_value}) is still elevated. Please call the office to speak to a clinician about your blood pressure: xxx-xxx-xxxx. We will also let your care team know. If you are experiencing symptoms, please call 911."
            - AND send 1st and 2nd BPs to NP
    - **If no input received to recheck prompt:**
        - ➔ After 1 hour, send a reminder text: "We haven't heard from you. It's important that you recheck and resubmit your BP within the hour ⌛**AND**
        - ➔ After 2 hours (window closes), send 1st HIGH BP to NP
- **If valid input ≤90 OR <50 received *[EXTREMELY LOW BP CONVERSATION]***
- "Your blood pressure ({systolic_value}/{diastolic_value}) is very low. Please wait 15 minutes, check it again, and let us know what your new BP is (Ex. 120/80)"
    - **Recheck: If valid input <180/110 received**
        - "{systolic/diastolic} has been recorded. It's good that your measurement has come up, please continue to monitor closely. If the top measurement is consistently less than 90 or the bottom measurement is consistently less than 50, please call the office to speak to a clinician about your blood pressure: xxx-xxx-xxxx. We will also let your care team know."
    - **Recheck: If valid input ≤90 OR <50 received**
        - ➔ "Your blood pressure ({systolic_value}/{diastolic_value}) is still quite low. Please call the office to speak to a clinician about your blood pressure: xxx-xxx-xxxx. We will also let your care team know. If you are experiencing symptoms, please call 911."
            - AND send 1st and 2nd BPs to NP
    - **If no input received to recheck prompt:**
        - ➔ After 1 hour, send a reminder text: "We haven't heard from you. It's important that you recheck and resubmit your BP within the hour ⌛
        - ➔ **AND**

After 2 hours (window closes), send 1st LOW BP to NP.

- **If input format incorrect**
  ➔ "I don't understand that blood pressure. Please send it as two numbers separated by a slash."
- **If no input received**
  - After 11 hours, send a reminder text: "We haven't heard from you. Please submit your BP as soon as possible."

PROGRAM FEEDBACK TO PARTICIPANT (BLOOD PRESSURE)

Arms: 1&2
Begin: 1st Saturday after 1st FULL week in the program
Repeats on days: Saturdays
At: 10:30am

Looks at last 3 BP Reminder responses:

- **If 3 of 3**
  - **If 3/3 within normal limits + support partner**
    - "You submitted 3 controlled BP measures this week. Well done! We've also let {{@partnerfirstname}} know. Keep up the good work!"
    - **If 3/3 within normal limits + no support partner**
    - "You submitted 3 controlled BP measures this week. Well done! Keep up the good work!"

  - **If 2/3 or 3/3 are HIGH + support partner**
    - "Good job taking your blood pressure X times this week! We noticed that X of your BP measurements this week were a little high. We may reach out to your provider or {{@partnerfirstname}} to help you manage your blood pressure. Please also reach out to them if you feel you need additional support."
    - **If 2/3 or 3/3 are HIGH + no support partner**
    - "Good job taking your blood pressure X times this week! We noticed that X of your BP measurements this week were a little high. We may reach out to your provider to help you manage your blood pressure. Please also reach out to them if you feel you need additional support."
  - **If 1/3 are HIGH**
    - "Good job taking your blood pressure {{@weeklybpquant}} times this week. We'll let {{@partnerfirstname}} know you're crushing this! This week, only one of your BP measures was high. There's room for improvement, but keep up the good work👌!"
- **If 1-2 of 3 Measurements submitted** (no qualitative feedback)
    - "You submitted X out of 3 BP measurements this week, thank you for monitoring. Next week, aim for 3 times!"
- **If 0 of 3 + support partner**
  - "We have not received any BP measurements from you this week, it's important that you monitor if your pressure is controlled. Don't forget to reach out to your support partner if you need help remembering. Questions? Call xxx-xxx-xxxx."
- **If 0 of 3 + no support partner**
  - "We have not received any BP measurements from you this week, it's important that you monitor if your pressure is controlled. Questions? Call xxx-xxx-xxxx."

  - If 3 out of last 10 measurements are not controlled (1st)

    - Send incident to nursing pool
    - Msg to Participant: Hi there PARTICIPANT_FIRSTNAME. It looks like you're struggling to keep your BP controlled, some of your measurements have been a little high lately. We've notified your Care Team and will step up your intervention and get your support partner involved 🖤
    - Here are three ways to help control your BP: 1) Keep your salt intake below 1,500mg (half a teaspoon) per day; 2) Take your medications every day as they were prescribed by your doctor; and 3) exercise regularly (about 30 mins/day or a long walk on most days of the week).

    - Msg to Partner: Hi there {{@partnerfirstname}}. PARTICIPANT_FIRSTNAME is struggling to keep their BP controlled, please talk to them about how important their health is to you and offer to provide support however you're able 🖤

  - If 3 out of last 10 measurements are not controlled (2nd)
    - Send incident to nursing pool
    - Msg to Participant: Hi there PARTICIPANT_FIRSTNAME. It looks like you're struggling to keep your BP controlled, some of your measurements have been a little high lately. We've notified your Care Team and will step up your intervention. You may hear from your care team by text message (from #215-650-8055) in the next few days 🖤
    - Sometimes it takes a little extra help to achieve BP control. For now, continue to take your meds as prescribed, limit your salt intake and exercise regularly. You got this 💪

  - Msg to Partner: Hi there {{@partnerfirstname}}. Hi there {{@partnerfirstname}}. PARTICIPANT_FIRSTNAME is still is struggling to keep their BP controlled, some of their measurements have been a little high lately. Your encouragement can really help; try letting them know how important their health is to you, and offer your support by promoting healthy activities like limiting salt and getting more exercise 🖤

  - If 3 out of last 10 measurements are not controlled (3rd+)
    - Send incident to nursing pool
    - Msg to Participant: Hi there PARTICIPANT_FIRSTNAME. Despite your best efforts, your blood pressure is still not controlled. We've notified your Care Team and we will step up your intervention. You may hear from your care team in the next few days about changes to your medications 🖤
    - Having high blood pressure can be dangerous in the long term, potentially causing stroke, kidney damage, or heart attack. To help prevent these, your care team may offer you more assistance in controlling your blood pressure through changing your prescriptions or adding additional medications. For now, continue to take your meds as prescribed, limit your salt intake and exercise regularly.

  - Msg to Partner: Hi there {{@partnerfirstname}}. PARTICIPANT_FIRSTNAME is still is struggling to keep their BP controlled. Additional medications may be recommended to help PARTICIPANT_FIRSTNAME get their BP controlled. You can help support them by encouraging them to speak with their doctor about their meds and other lifestyle changes that can help 💊

- If pts submit BPs outside of the standard windows, it does not contribute weekly feedback logic, however it will be handled as follows:
  - **If valid input <180/110 received and >=90/50**

        - Good job taking your BP! You will still need to submit a BP measurement when next prompted.
    - **If valid input ≥180/110 received**
        - ***[SEVERELY ELEVATED CONVERSATION & Incident to Nurse Practitioner]***

If valid input <90 or <50 received

[EXTREMELY LOW CONVERSATION & Incident to Nurse Practitioner]

    - **If input format incorrect**
        - "I don't understand that blood pressure. Please send it as two numbers separated by a slash."

MEDICATION ADHERENCE

RANDOMIZED AUTOMATED REMINDERS

Arms: 1&2
Begin: 1st Thursday in program
Repeats on days: Thursdays
At: 8am

- **Participant will receive one of:**
    - "Remembering to take your medications is easier when you get into a habit.
      OR
    - "Some people use a pill sorter to keep track of their medications.
      OR
    - "Every day is a new opportunity to take control of your health.
      AND
      →"How many days out of the last 7 days did you take all your BP medication(s) as prescribed? Please input a single number 0-7"

PROGRAM FEEDBACK TO PARTICIPANT (MEDICATION)

Arms 1&2:
Begin: 1st Thursday in the program
Repeats on days: Thursdays
At: 7pm (if no response)

After 11hours send reminder text:

"Just a reminder to text us about your medications. How many days out of the last 7 days did you take all of your BP medication(s) as prescribed? Please input a single number 0-7."

At: upon response to Medication Adherence text:

- **If 7 of 7:**
    - Well done! You reported taking your BP meds 7 of the last 7 days. Taking your medication as prescribed every day is the best way to control your BP.
        -

- **If 4-6 of 7, respond**
    - ***[MEDICATION DIFFICULTY CONVERSATION]*** "Thank you. You reported taking your BP meds X of the last 7 days You're definitely on the right track, but we're hoping to see 7 out of 7 consistently!.. Let us know what might be causing you difficulty by responding with a number 1 – 5:
        - 1) I need a refill
        - 2) I can't remember to take my meds
        - 3) I'm taking them, but not as prescribed
        - 4) Other
        - 5) No difficulty, I can do better
            - If Responds with: **1) I need a Refill**
                - Keeping your medications filled is important so that you don't miss doses. First, check with your pharmacist to see if they can refill it. Often prescriptions can be mailed to your home. If your prescription has expired, please contact your care team at xxx-xxx-xxxx
            - Responds with **2) I can't remember to take my meds**
                - "It can be helpful to use a pill sorter or keep track of your medications with a calendar. You can use your phone or a digital watch for alarms. You can do this!"
            - Responds with **3) I'm taking them, but not as prescribed**
                - "It's important to take your medications as prescribed. If you're experiencing side effects, having trouble affording your medications or another issue, reach out to your provider's office (xxx-xxx-xxxx) to discuss ways that they may help you."
            - Responds with **4) Other**.
                - "Sometimes it can be hard to take our medications as prescribed. Can you tell us in just a few words what you're struggling with?
                - Ok thank you for letting us know, it's important for us to understand what difficulties our patients are facing. Please also be sure to call your provider's office (xxx-xxx-xxx) to see if they can help you with this difficulty."

- **If 0-3 of 7**
    - ***[CONVERSATION]*** "Thanks for that information. You reported taking your BP meds X of the last 7 days. It looks like you might be struggling a little bit with your medications. Let us know what might be causing you difficulty by responding with a number, 1-5:
        - 1) I need a refill
        - 2) I can't remember to take my meds
        - 3) I'm taking them, but not as prescribed
        - 4) Other
        - 5) No difficulty, I can do better
            - Responds with: **1) I need a Refill**
            - "Keeping your medications filled is important so that you don't miss doses. First, check with your pharmacist to see if they can refill it. Often prescriptions can be mailed to your home. If your prescription has expired, please contact your care team at xxx-xxx-xxxx

- Responds with **2) I can't remember to take my meds**
  - o "It can be helpful to use a pill sorter or keep track of your medications with a calendar. You can use your phone or a digital watch for alarms. You can do this!"
- Responds with **3) I'm taking them, but not as prescribed**
  - o "It's important to take your medications as prescribed. If you're experiencing side effects, having trouble affording your medications or another issue, reach out to your provider's office (xxx-xxx-xxxx) to discuss ways that they may help you."
- Responds with **4) Other.**
  - o "Sometimes it can be hard to take our medications as prescribed. Can you tell us in just a few words what you're struggling with?
  - o Ok thank you for letting us know, it's important for us to understand what difficulties our patients are facing. Please also be sure to call your provider's office (xxx-xxx-xxx) to see if they can help you with this difficulty."

- **If input format incorrect**
  - "Sorry, we do not understand that response. Please input a number: 0-7. Questions? Call xxx-xxx-xxxx."

- **If no input received**
  - "You did not report taking your medication at all this week. Please remember to monitor your BP and keep up taking your meds as prescribed next week. Remember to respond to our prompts and let us know about your medications every week. Questions? xxx-xxx-xxxx or email Xxxx@pennmedicine.upenn.edu.

FACILITATED PARTNER FEEDBACK TO PARTICIPANT (MEDICATION)

Arms: 1&2 (participants)
Begin: 2nd Friday from participant start
Repeats: weekly on Friday, if support partner active and no opt-out from partner
At: 11:15AM

Looks back at participant medication adherence response

If Meds = 7
Participant will receive one of:

- "{{@partnerfirstname}} wants you to know they think you're doing a great job taking your BP medications everyday as prescribed! 🎉"
- {{@partnerfirstname}} sends 👍👍 for taking your BP meds every day as prescribed!
- You're on 🔥 {{@partnerfirstname}} says keep up the great work taking your BP meds every day as prescribed.

If Meds = 5-6:
Participant will receive one of:

- {{@partnerfirstname}} says, you're so close! Next week aim to take your BP medications as prescribed every day.

- {{@partnerfirstname}} says it's really important to them that you take your BP meds as prescribed every day 🖤 You can do this!
- Your health is super important to {{@partnerfirstname}}, and they want you to aim to always take your BP meds all 7 days as prescribed 🏆

If Meds 0-4:
Participant will receive one of:

- {{@partnerfirstname}} says everyone forgets once in a while, but you can lean on me to help you with taking your BP medications as prescribed.
- {{@partnerfirstname}} cares about your health and is here to support you if you need help with taking your BP medications as prescribed.

If Meds = Missing (no response from participant)
Participant will receive one of:

- {{@partnerfirstname}} asks you to please work on taking your BP meds as prescribed every day. It's important to them that you get your blood pressure under control because they care about your health.
- {{@partnerfirstname}} cares about your health and asks you to please work on taking your BP meds as prescribed every day. They are here to support you if you need help 🖤

PARTNER SUPPORT

Partners nominated by patients in Arms 1&2 will be called and their participation requested.

AFTER ENROLLMENT

Arms: 1 & 2 (to partner only)
Begin: Day 0
Repeats: does not repeat
At: upon enrollment

"Welcome to SupportBP! Thank you for participating in support of <participant_name>. You'll receive updates about <participant_name>'s performance on Thursdays and Saturdays. Questions? Please call xxx-xxx-xxxxor email SupportBP@uphs.upenn.edu."

"Please be advised that standard SMS texting is not secure, so other people may be able to see the information in these messages. Text "bye" at any time to stop participating in this program. "

Arms 1&2 (to participant only)
Begin: upon partner enrollment
Repeats: does not repeat

Hello PARTICIPANT_FIRSTNAME! {PartnerFirstName} has agreed to be your support partner throughout the SupportBP program. They will be updated on your progress and encouraging you to stay on track with treatments.

BLOOD PRESSURE

PROGRAM FEEDBACK TO PARTNER (BLOOD PRESSURE)

Begin: 1st Saturday after 1st FULL week in the program
Repeats on days: Saturdays
At: 10:30am

Looks at patients' last 3 BP Reminder responses:

- **If 3 of 3 submitted, all 3 controlled**
    - "<PATIENT NAME> submitted all 3 readings this week and all 3 were controlled. Be sure to let them know what a great job they're doing tracking their BP regularly If 3 of 3 submitted and 1 not controlled
        - **PARTICIPANT_FIRSTNAME submitted all 3 readings this week, and one was not under control. There's some room for improvement, but be sure to let them know what a great job they're doing tracking their BP regularly**
    - **If 3 of 3 submitted and 2+ are not controlled**
        - **PARTICIPANT_FIRSTNAME submitted all 3 readings this week, and some of them were not controlled. Reach out to them to see if they could use your support, but also be sure to let them know what a great job they're doing tracking their BP regularly**
- **If 1-2 of 3**

        - “<PATIENT_NAME> submitted X out of 3 BP measurements this week. They may need your help remembering to take their BP. Thanks for your support!”
- **If 0 of 3**
    - “We have not received any BP measurements from <PATIENT_NAME> this week. Reach out and see if they need any help remembering to take their BP. Thanks for your support!”

MEDICATION ADHERENCE

PROGRAM FEEDBACK TO PARTNER (MEDICATION) Arms: 1&2

Begin: 1st Friday in program
Repeats on days: Fridays
At: 8am

Looks at last Medication Adherence response:

- **If 7 of 7, respond**
    - “<PATIENT_NAME> took their BP meds as prescribed 7 of 7 times this past week. We’ll let them know you think they’re doing great, unless you text “No” in reply to this message within the next 2 hours.”
-
- **If 0-6 of 7**
- PARTICIPANT_FIRSTNAME reported taking their BP meds as prescribed {{@daysadherent}} of the last 7 days. We'll let them know you think it's important they always take their meds as prescribed and that you’re available to help, unless you text "No" in reply to this message within the next 2 hours.
- **If no input received**
    - “We did not receive any info from PARTICIPANT_FIRSTNAME about taking their BP meds the last 7 days. We’ll let them know you think it’s important they take their meds as prescribed, unless you text “No” in reply to this message within the next 2 hours. They may also need your help to do this, please reach out to them and see what they may find helpful.
        - If support partner responds to any of the above with “No,” then respond,
            - “Thank you, we will not send a message to <participant name> today on your behalf. Please be sure to support <participant name> in regular BP monitoring and daily medication adherence.”
        - If support partner responds to any of the above with invalid input, then respond,
            - “Sorry, we do not understand that response. Please only respond with “No” if you do not want us to send a message to <participant>. Otherwise, no response is necessary. Questions? Call xxx-xxx-xxxx.”

END OF PROGRAM

FINAL MESSAGING

ARMS: 1&2 PATIENTS & SUPPORT PARTNERS
Begin: 6M from participant start
Repeats on: does not repeat
At: 8am

PATIENTS

- “Congratulations on completing your time in SupportBP! Thank you for your participation. We hope you found it helpful in controlling your BP! You will no longer receive blood pressure reminders or feedback. Questions? Please call xxx-xxx-xxxxor email Xxxx@pennmedicine.upenn.edu.”

SUPPORT PARTNERS

- “Thank you for your participation in Support BP! <PATIENT_NAME>’s participation has ended. You will no longer receive text messages from us. Questions? Please call xxx-xxx-xxxxor email Xxxx@pennmedicine.upenn.edu.”

RANDOMIZED LIFESTYLE MESSAGING

Physical Activity

ARMS: 1&2, Patients
Begin: 1st Monday from participant start
Repeats on: every 6th Monday
At: 1pm

Patient receives one of:

- Control Your BP Tip #17: While any aerobic activity (walking, jogging, dancing) does the heart good, try to find something you enjoy doing. This makes it easier to commit to a regular routine and motivates you to get up and moving.
- Control Your BP Tip #34: Getting enough physical activity is all about what works best for YOU! Learn more here: https://www.youtube.com/watch?v=0i1lCNHaxhs&feature=youtu.be
- Control Your BP Tip #68: A good starting goal for exercise is at least 150 minutes a week, but don't want to sweat the numbers, just move more! Find forms of exercise you like, and build more opportunities to be active in your routine.

Med Adherence

ARMS: 1&2, Patients
Begin: 2nd Tuesday from participant start
Repeats on: every 5th Tuesday
At: 8am

- Patient receives one of:
  - Control Your BP Tip #1: It's important to take medications exactly as prescribed and report any side effects to your doctor. Share a complete list of all the medications you take, including the over-the-counter drugs and supplements with your doctor.
  - Control Your BP Tip #5: Take medications at the same time each day along with other daily events, like brushing your teeth.
    Control Your BP Tip #10: To help you remember to take your BP meds as prescribed, use a weekly pill box with separate sections for each day or time of day.
  - Control Your BP Tip #20: Ask family and friends to help remind you to take your medications.

- Control Your BP Tip #40: Leave notes to remind yourself to take your medications, or use your smart phone calendar or watch with alarm to help you remember.
- Control Your BP Tip # 7: Ask your doctor or pharmacist before buying new over-the-counter medicinea, such as an antihistamine, "cold tablets," or vitamin supplements to be sure that they won't interfere with your prescribed medicine.
- Control Your BP Tip #14: Always get your prescription filled on time, so you don't run out.
- Control Your BP Tip #21: Don't take more of your medicine than the prescribed dose.
- Control Your BP Tip #33: Always check with your doctor before you stop taking a medicine.

DIet

ARMS: 1&2, Patients
Begin: 3rd Wednesday from participant start
Repeats on: every 5th Wednesday
At: 8am

Patient receives one of:

- Control Your BP Tip #2: Cut back on the amount of salt (sodium) that you consume. The latest Dietary Guidelines recommend consuming less than 2,300 mg of sodium/day - less than a teaspoon of salt. For people with high blood pressure, less than 1,500 mg of sodium/day is best.
- Control Your BP Tip #13: Read food labels. Look for "low salt" or "low sodium" versions of the food and beverages you normally buy.
  Control Your BP Tip #25: Try to eat fewer processed foods. Nearly 80% of the sodium we eat comes from processed, prepackaged, and restaurant foods.
- Control Your BP Tip #37: Instead of adding salt to your foods, try using salt substitutes such as spices, garlic, herbs, and other seasonings in place of some or all of the salt to add flavor.
- Control Your BP Tip #49: Potassium helps your body get rid of sodium and eases tension in your blood vessel walls, lowering BP. Incorporating foods rich in potassium (like spinach, bananas and sweet potatoes) into your diet can improve heart health, but talk with your doctor about the potassium level that's right for you, especially if you have significant kidney disease.
- Control Your BP Tip #52: Delicious, simple, affordable, and quick! Here are some recipes that are good for your heart and your wallet: https://recipes.heart.org/en/recipes
- Control Your BP Tip #66: Check out this list of heart healthy recipes! https://millionhearts.hhs.gov/learn-prevent/recipes.html
- Control Your BP Tip #44: Eating out? Controlling your sodium intake by ordering carefully: Choose fresh greens and fruits. Ask for oil and vinegar for your salad or ask for dressing on the side. Request that your dish be prepared without added salt. Remember portion control. You can always bring home a to-go box!
- Control Your BP Tip #31: Watch out for the "Salty 6" that add the most salt to your diet. Read labels and choose the lowest level of sodium you can find for: breads and rolls, cold cuts and cured meats, pizza, poultry, soup, and sandwiches.
- Control Your BP Tip #48: Alcohol contains many calories and sugar, which can contribute to increased body fat and weight gain - factors that can lead to higher blood pressure. Also, alcohol can reduce the effectiveness of many BP meds.
- Control Your BP Tip #55: The DASH eating plan is guaranteed to help lower blood pressure in as fast as 2 weeks! Learn more here: https://tinyurl.com/y8apyobh

General

ARMS: 1&2, Patients
Begin: 4th Thursday from participant start
Repeats on: every 5th Thursday
At: 8am

Patient receives one of:

- Control Your BP Tip #3: Ways to lower your blood pressure naturally: 1. Regular physical activity. 2. Eat less salt. 3. Add more potassium to your diet. 4. Limit alcohol. 5. Reduce your stress.
- Control Your BP Tip #16: Learn more about how to treat high blood pressure: https://www.youtube.com/watch?v=XbLmIoyDJuE
- Control Your BP Tip #26: Along with eating right and being active, real health includes getting enough sleep, practicing mindfulness, managing stress, keeping your mind and body fit, and connecting socially.
- Control Your BP Tip #30: Blood pressure is the force of your blood moving against the walls of your arteries. Download this to learn more and get more tips on lifestyle changes that can help lower your blood pressure: https://tinyurl.com/y2orn228
- Control Your BP Tip #51: Knowing your blood pressure and successfully controlling it depends on correct readings. Download this fact sheet for steps to make sure the BP numbers you record at home are right: https://tinyurl.com/y2ylsv4y
- Control Your BP Tip #57: Small changes can make a BIG difference in your BP control. Find out more here: https://tinyurl.com/y26cyngs
- Control Your BP Tip #61: Learn more about taking your blood pressure: https://tinyurl.com/yxbszd4e

Stress Management

ARMS: 1&2, Patients
Begin: 5th Friday from participant start
Repeats on: every 5th Friday
At: 8am

Patient receives one of:

- Control Your BP Tip #9: Stress can cause your blood pressure levels to spike, so make sure to do things that help you relax and reset like listen to your favorite music, go for a walk or take a yoga class.
- Control Your BP Tip #11: Reframe your mindset. Focus on things you can control, instead of worrying about situations you can't. Many anxieties stem from "what if" - instances that might not ever occur. Remind yourself to stay present to calm worries.
- Control Your BP Tip #29: Avoid stress triggers. Try to avoid putting yourself in unnecessary stressful situations. For example, try leaving for work a few minutes early to beat rush-hour traffic.
- Control Your BP Tip #45: Practice gratitude. Acknowledging all life's possibilities helps to shift the focus away from what we are lacking. Also, outwardly expressing gratitude to others can help reduce feelings of stress.

- Control Your BP Tip # 54: Take time to relax and do things that bring you joy. Whether that's spending time with loved ones or listening to music or a podcast on your commute, find time to incorporate small moments of happiness.

[BP MONITORING TUTORIAL]
Begins when participant texts the word SUPPORT at any time, or in response to welcome message question about taking BP measurements correctly, or if participant replies “1” to the non-engagement conversation (below)

1. BP Monitoring Tutorial. This will take about 5 minutes to walk you through the process of correctly taking your blood pressure, one step at a time. At the end, you can submit a practice measurement. Reply with the word NEXT when you have your blood pressure cuff nearby.
2. First, make sure you avoid drinking caffeine, smoking, and exercise in the 30 minutes prior to taking your blood pressure, and be still for at least 5 minutes before you begin. Reply NEXT when you’re ready for the next instruction.
3. Sit up straight in a firm chair with your back supported, and where both feet can be flat on the floor. Rest your LEFT arm comfortably at about chest level. Reply NEXT for the next instruction.
4. Insert your bare arm into the cuff so that the cord exits the cuff towards your wrist. Place the cuff above the bend in your elbow. Pull the extra slack on the cuff and fasten using the Velcro. Not too tight! You should be able to comfortably insert a finger between your arm and the cuff. Reply NEXT when you’re ready for the next instruction.
5. Almost there! You can press the big blue button to start the measurement. Remain quiet, and avoid moving and using devices while the measurement is in progress. The cuff will automatically inflate and tighten around your upper arm. It should take less than a minute to display your blood pressure. Reply NEXT for the next instruction.
6. To report your blood pressure to us, send us the larger number followed by the smaller number, separated with a slash, like this: 141/89. Send us your practice measurement now (don’t worry, we won’t count this one)!
7. [When BP received]: That’s it, great job! If you feel like you need additional assistance, reply MORE. Otherwise, you’re ready to get started! If you need to repeat this again in the future, text SUPPORT at any time and the tutorial will start over.
8. [If MORE]: Ok, we’re sorry about that. A program coordinator will reach out to you within the next business day to help.
    a. AND, send incident to RC to follow-up with participant.

Non-Engagement

Begins when participant has not engaged in any way with texting for at least 7 consecutive events (approximately 2 weeks)

Hi there PARTICIPANT_FIRSTNAME, it's been almost 2 weeks since we've heard from you ☹ We're reaching out because staying accountable to regular blood pressure monitoring is one of the best ways to manage your hypertension. Is there anything we can help with to get you back to the program? Please reply 1, 2 or 3:

1. Yes, I need help with using my monitor/taking my blood pressure
    a. If 1: [trigger BP Monitoring Tutorial]

2. No, I just got busy. I will send in a measurement now.
    a. If 2: That's great, way to get back on track!
3. It's something else
    a. If 3: Ok, can you tell us a little more about what that is?
    b. If reply: Ok, thanks for letting us know. A program coordinator will reach out to you within the next business day to try to help you with that!
    c. AND, send incident to RC to follow-up with participant

Appendix C: Stepped Escalation Intervention Flow Diagram

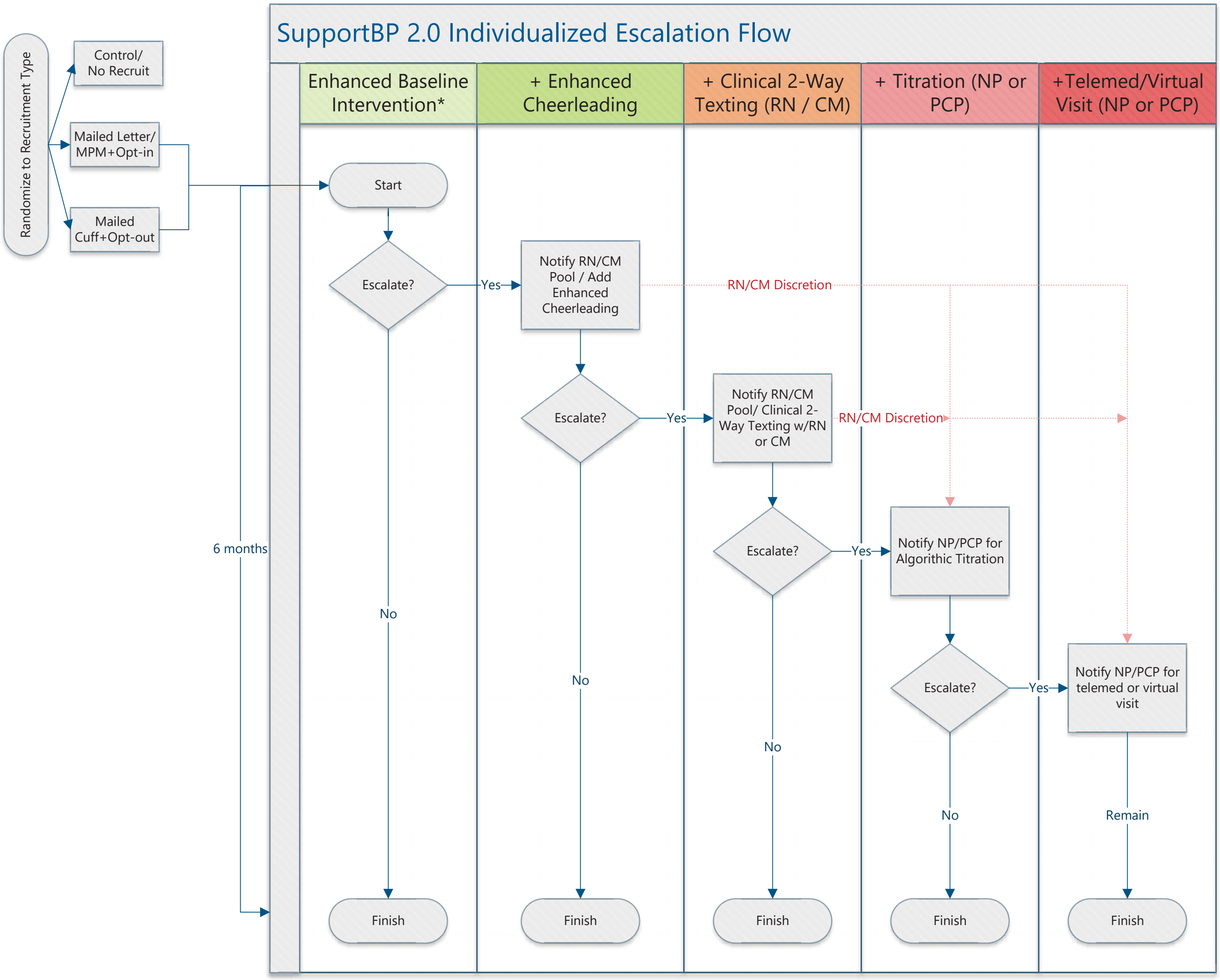

*** Enhanced Baseline Intervention:** BP prompts x 3/wk, increased granularity around med adherence, positive feedback on BP measurements ("You've submitted three controlled measurements in a row! Great job!"), lifestyle messaging (diet, exercise, self-efficacy, reduce stress, etc), feedback and notice of escalation to PCP(or "You've submitted 3 elevated measurements ina row, we will notify your Dr and step up your intervention")

*** Enhanced Cheerleading:** Baseline + Share BP measurement/adherence specifics with Partner, stronger remote engagement (names, personal message?), encourage outside engagement with participant, share same feedback/milestones with partner (<name> has submitted 3 controlled measurements in a row!")

***Clinical 2Way Texting:** Baseline + Cheerleading + asynchronous text communication with RN or CM team for clarity, coaching, symptom clarification. Directly from patient record.

***Titration:** Baseline + Cheerleading +Clinical 2way Texting + medication changes. By this point in intervention enough info regarding adherence, lifestyle, coaching, etc should be available to justify increasing dosage or adding/changing medications.

*** Virtual Visit:** Baseline + Cheerleading +Clinical 2way Texting + Titration + virtual provider visit. If titration fails after defined period, virtual visit for further clinical workup/ referral/additional med changes/etc. Patient will have visit and remain in this phase, which incorporates all steps of the intervention.

RN or CM notified of escalations through steps 1-3. At any point of escalation to next step, RN/CM may decide to directly escalate to NP/PCP for titration or virtual visit at his/her discretion. Steps 4 and 5, **PCP or NP** notified of escalations for med management/further clinical evaluation.

Appendix D: Final Interview Guide

<u>Plan for Interview:</u> Because each participant has a different level of engagement with the program, we will need to look at each individual, refer to RedCap and WayToHealth to know which interventions they participated in, and use this information to decide which categories of questions to ask. A checkbox will be created listing the various levels of support that participants used (or did not use) in order to determine which set(s) of questions will be asked during the interview.

<u>High BP Participation/High Care Team Engagement</u>
These patients submitted minimum # of BPs within the first/last 6 weeks of the program, and engaged with the clinical team.

Introduction

Hello, my name is (research staff name). I am calling from the Support BP Program at Penn Medicine. Is this _____?

We are conducting a brief interview to learn more about your most recent experience with your care team at Penn Family Care in the management of your hypertension (high blood pressure). It will take approximately 20-30 minutes to complete this interview and you will receive $50 for your time to complete it. Are you willing to participate?

If yes,

Thank you for agreeing to participate! This call will be recorded to accurately keep track of the feedback you provide, but your responses will be confidential. Loss of confidentiality is possible, but unlikely. We will minimize this risk by using de-identified information and by maintaining all information on Penn Medicine's secure drive. You may stop the interview at any time without any consequence to you. Do you agree to this phone call being recorded?

If no,

Thank you so much for your time, and have a wonderful rest of your day.

If yes,

The recording will begin now. Thank you so much for agreeing to participate in this interview.

Background/ Questions

You were asked to participate in the Support BP Program because we were testing ways to help people improve their health and better manage their blood pressure. With the help of your care team at Penn Family Care, we asked you to submit blood pressure measurements over the course of 6 months to monitor and help in managing your health.

- When you were enrolled in SupportBP, the welcome text message read *"PARTICIPANT_FIRSTNAME, your SupportBP program starts today! You can expect to receive a text to measure and submit your blood pressure (BP) three times each week, and a text about how you're taking your medications once a week. We will share your BP measurements with your care team, and add additional support to your program if you need it, as we go. We hope you find*

this program helpful! Call us if you have any questions: xxx-xxx-xxxx." How did you feel about this text message?

  - Was this information clear and did you have a smooth transition into the program or is there something you'd change?
  - How did you feel about receiving instructions on how to get started with taking your BP after the welcome text? Would you have preferred to have receive instructions immediately with the welcome text?
- Did the welcome text make expectations about your participation in the study clear? How could we have made this better?
- Did you need further instructions on how to take or submit your blood pressure to the program? Would you say this process was easy/straightforward or confusing/difficult? If the latter, can you describe your difficulties?

Intervention Questions

Intervention: Text messaging

- One of the program interventions, as you know, was text messaging. You received 4 text message prompts each week, asking for information about your blood pressure and how often you took your blood pressure medication as prescribed by your doctor. You also received a series of tips and facts about blood pressure, as well as feedback from the program regarding your blood pressure control. *I'm going to be sending you a series of example messages we sent you, and asking you a few questions about each:*
  - [Send bp measurement prompt] This message was sent three times each week. How did you feel about how often you had to send in your BP measurements? Would you have preferred a different frequency?
  - Which days would you prefer to send in your BP measurements? Can you tell me about your preferences and why? These messages were normally sent at 8am- is there a time that would have worked better for you? Why is that?
  - [Send lifestyle tip message & medication adherence text] How have the reminders about taking your medications and messages about lifestyle changes impacted your management of your hypertension? Since the messages have stopped, are you still checking your BP regularly? Did any of the tips help you to implement any lifestyle changes towards managing your BP?
  - [Send a feedback text, maybe one positive/one negative] You may have received one or both of these messages during your participation. What are your thoughts on the content of the messages? Would you have preferred for the feedback to be more encouraging, for example?

Intervention: Social support

- We asked you for the names of up to 3 partners to support and encourage you throughout the program. The purpose was to add your support partner(s) to your program if your blood pressure measurements routinely exceeded guidelines. If added, they would have received weekly updates about your medication adherence and blood pressure monitoring, and would have be notified if your blood pressures were routinely elevated above a certain threshold.
  - Support Partner (listed and used)

        - How did you feel about the social support you received while in this program? Did you communicate with your support partner about your hypertension outside of this program?
        - What would you like to change about messaging you received from your support partner? (ex: general message vs personalized messaging [in the future])
        - Were you comfortable with the idea of having a support partner? Why or why not?
        - Outside of this program, did you have other people in your life to support you with your hypertension management?
            - *If yes:* How did they help you? (ex, conversations at home, other messages etc)
            - *If no:* Would you have liked to have someone outside of your Penn Medicine care team support you in your everyday life?
    - Support Partner (listed and not used)
        - We noticed that you listed a support partner (or partners) who we were not able to get in touch with. How do you think your program may have been different had we been able to enroll one of your support partners?
        - Outside of this program, did you have other people in your life to support you with your hypertension management?
            - *If yes:* How did they help you? (ex, conversations at home, other messages etc)
            - *If no:* Would you have liked to have someone outside of your Penn Medicine care team support you in your everyday life?

    - Support Partner (not listed)
        - We noticed that you did not list a support partner (or partners), is there a reason why?
        - Were you comfortable with the idea of having a support partner?
        - Outside of this program, did you have other people in your life support you with your hypertension management?
            - *If yes:* How did they help you? (ex, conversations at home, other messages etc)
            - *If no:* Would you have liked to have someone outside of your Penn Medicine care team support you in your everyday life?
        - What could we have done to make having a support partner better/easier?

Intervention: Clinical 2-way texting

- As a reminder, your care team was able to contact you via the study text-messaging platform to obtain additional information, provide additional guidelines for the management of your blood pressure, or set up a phone consultation or office visit.
    - Currently, text messaging from providers is not the norm, so how did you feel about receiving a text message from your care team?
    - Was it difficult to keep up with the messaging you were receiving from your clinical care team?

- Did you trust the messages? Why or why not?
- Did you find the advice/information shared from the clinical team helpful/useful?
- Although the person texting you is a provider at Penn Family Care and working in collaboration with your personal physician, they may not have been your personal physician- what are your thoughts about receiving care from a team like this, or from someone who is not normally involved in your regular care?
- Is there something else you would have preferred for them to have done? Information to have given you?
- Did you have a preference for texting or using MyPennMedicine with your provider? Why is ___ method preferred?

Intervention: Medication adjustment

- Your care team may have changed the dosing of your existing medications, changed your medications, or prescribed additional medications to help manage your blood pressure. Keeping in mind that I am not a physician, I'm going to ask you some questions about having your medication adjusted:
  - Did you implement the medication change(s) suggested by your care team? If no, why not?
  - Was it an easy process? What concerns did you have, if any? And were they addressed?
  - How did you feel about having your medications changed multiple times?

Intervention: Visit/engagement with care team

- Your care team may have requested a virtual or in-person office visit to follow-up on your medications and perform additional diagnostics to help manage your blood pressure.
  - During the duration of your enrollment, did you have an in person or virtual visit with either your provider or another member of your care team for management of your hypertension?
  - Did you have a preference for virtual or in person visits? Tell me about your preference and why.
  - During the duration of your enrollment, did you schedule more frequent visits with your PCP? Did the program influence how often you scheduled visits with your PCP? (If yes) Tell me more about this.

Ending Questions

- In what ways did you find remote monitoring most helpful?
- What could we have done differently? What additional steps could we have taken to help you?
- Was it clear, at each point of the study, what was expected of you? If not, what did you need more help with?
- Did you notice any changes in your habits/lifestyle management due to this study? examples/why/why not?
- Would you have preferred the length of the remote monitoring program be different? In what way? Please describe.
- On a scale of 1-10, how likely are you to recommend the remote monitoring program to a friend or family member who may need this?

- Is there anything else you'd like to share with us about the program, the way you manage your blood pressure, or how we could have improved upon your program?

<u>Low BP Engagement/Mixed Clinical Team Engagement</u>
These patients submitted less than the minimum number of BPs *OR* submitted the minimum outside of the first/last 6 weeks. These patients may or may not have engaged with the Care team.

Introduction

Hello, my name is (research staff name). I am calling from the Support BP Program at Penn Medicine. Is this _____?

We are conducting a brief interview to learn more about your most recent experience with your care team at Penn Family Care in the management of your hypertension (high blood pressure). It will take approximately 20-30 minutes to complete this interview and you will receive $50 for your time to complete it. Are you willing to participate?

If yes,

Thank you for agreeing to participate! This call will be recorded to accurately keep track of the feedback you provide, but your responses will be confidential. Loss of confidentiality is possible, but unlikely. We will minimize this risk by using de-identified information and by maintaining all information on Penn Medicine's secure drive. You may stop the interview at any time without any consequence to you. Do you agree to this phone call being recorded?

If no,

Thank you so much for your time, and have a wonderful rest of your day.

If yes,

The recording will begin now. Thank you so much for agreeing to participate in this interview.

Background/ Questions

You were asked to participate in the Support BP Program because we were testing ways to help people improve their health and better manage their blood pressure. With the help of your care team at Penn Family Care, we asked you to submit blood pressure measurements over the course of 6 months to monitor and help in managing your health.

- When you were enrolled in SupportBP, the welcome text message read *"PARTICIPANT_FIRSTNAME, your SupportBP program starts today! You can expect to receive a text to measure and submit your blood pressure (BP) three times each week, and a text about how you're taking your medications once a week. We will share your BP measurements with your care team, and add additional support to your program if you need it, as we go. We hope you find this program helpful! Call us if you have any questions: xxx-xxx-xxxx."* How did you feel about this text message?
  - Was this information clear and did you have a smooth transition into the program or is there something you'd change?

    - How did you feel about receiving instructions on how to get started with taking your BP after the welcome text? Would you have preferred to have receive instructions immediately with the welcome text?
- Did the welcome text make expectations about your participation in the study clear? How could we have made this better?
- Did you need further instructions on how to take or submit your blood pressure to the program? Would you say this process was easy/straightforward or confusing/difficult? If the latter, can you describe your difficulties?

Reduced Engagement

- Our goal is to help future participants engage in a program like this and we'd like to hear your feedback. We noticed that you responded to the prompts in the beginning of the program but did so less frequently towards the end of the 6-month window. Can you tell me about this?
    - What changed between when you consented/ started the program and when you completed the program?
    - Were there any specific levels of support that influenced your participation? For example, when your care team wanted to change your medication, or have you come into the office for a BP check?
    - What, if any, outside factors influenced your participation?
- Can you tell me about your feelings about the program in the beginning compared to the end?

Ending Questions

- In what ways did you find remote monitoring most helpful?
- What could we have done differently? What additional steps could we have taken to help you?
- Was it clear, at each point of the study, what was expected of you? If not, what did you need more help with?
- Did you notice any changes in your habits/lifestyle management due to this study? examples/why/why not?
- Would you have preferred the length of the remote monitoring program be different? In what way? Please describe.
- On a scale of 1-10, how likely are you to recommend the remote monitoring program to a friend or family member who may need this?
- Is there anything else you'd like to share with us about the program, the way you manage your blood pressure, or how we could have improved upon your program?

<u>High BP Engagement/No Clinical Care Team Engagement</u>

These patients submitted the minimum number of BPs within the first/last 6 weeks of program but did not engage with the care team at all.

Introduction

Hello, my name is (research staff name). I am calling from the Support BP Program at Penn Medicine. Is this _____?

We are conducting a brief interview to learn more about your most recent experience with your care team at Penn Family Care in the management of your hypertension (high blood pressure). It will take approximately 20-30 minutes to complete this interview and you will receive $50 for your time to complete it. Are you willing to participate?

If yes,

Thank you for agreeing to participate! This call will be recorded to accurately keep track of the feedback you provide, but your responses will be confidential. Loss of confidentiality is possible, but unlikely. We will minimize this risk by using de-identified information and by maintaining all information on Penn Medicine's secure drive. You may stop the interview at any time without any consequence to you. Do you agree to this phone call being recorded?

If no,

Thank you so much for your time, and have a wonderful rest of your day.

If yes,

The recording will begin now. Thank you so much for agreeing to participate in this interview.

Background/ Questions

You were asked to participate in the Support BP Program because we were testing ways to help people improve their health and better manage their blood pressure. With the help of your care team at Penn Family Care, we asked you to submit blood pressure measurements over the course of 6 months to monitor and help in managing your health.

- When you were enrolled in SupportBP, the welcome text message read *"PARTICIPANT_FIRSTNAME, your SupportBP program starts today! You can expect to receive a text to measure and submit your blood pressure (BP) three times each week, and a text about how you're taking your medications once a week. We will share your BP measurements with your care team, and add additional support to your program if you need it, as we go. We hope you find this program helpful! Call us if you have any questions: xxx-xxx-xxxx."* How did you feel about this text message?
    - Was this information clear and did you have a smooth transition into the program or is there something you'd change?
    - How did you feel about receiving instructions on how to get started with taking your BP after the welcome text? Would you have preferred to have receive instructions immediately with the welcome text?
- Did the welcome text make expectations about your participation in the study clear? How could we have made this better?
- Did you need further instructions on how to take or submit your blood pressure to the program? Would you say this process was easy/straightforward or confusing/difficult? If the latter, can you describe your difficulties?

Intervention Questions

Intervention: Text messaging

- One of the program interventions, as you know, was text messaging. You received 4 text message prompts each week, asking for information about your blood pressure and how often you took your blood pressure medication as prescribed by your doctor. You also received a series of tips and facts about blood pressure, as well as feedback from the program regarding your blood pressure control. *I'm going to be sending you a series of example messages we sent you, and asking you a few questions about each:*
  - [Send bp measurement prompt] This message was sent three times each week. How did you feel about how often you had to send in your BP measurements? Would you have preferred a different frequency?
  - Which days would you prefer to send in your BP measurements? Can you tell me about your preferences and why? These messages were normally sent at 8am- is there a time that would have worked better for you? Why is that?
  - [Send lifestyle tip message & medication adherence text] How have the reminders about taking your medications and messages about lifestyle changes impacted your management of your hypertension? Since the messages have stopped, are you still checking your BP regularly? Did any of the tips help you to implement any lifestyle changes towards managing your BP?
  - [Send a feedback text, maybe one positive/one negative] You may have received one or both of these messages during your participation. What are your thoughts on the content of the messages? Would you have preferred for the feedback to be more encouraging, for example?

Intervention: Social support

- We asked you for the names of up to 3 partners to support and encourage you throughout the program. The purpose was to add your support partner(s) to your program if your blood pressure measurements routinely exceeded guidelines. If added, they would have received weekly updates about your medication adherence and blood pressure monitoring, and would have be notified if your blood pressures were routinely elevated above a certain threshold.
  - Support Partner (listed and used)
    - How did you feel about the social support you received while in this program? Did you communicate with your support partner about your hypertension outside of this program?
    - What would you like to change about messaging you received from your support partner? (ex: general message vs personalized messaging [in the future])
    - Were you comfortable with the idea of having a support partner? Why or why not?
    - Outside of this program, did you have other people in your life support you with your hypertension management?
      - *If yes:* How did they help you? (ex, conversations at home, other messages etc)
      - *If no:* Would you have liked to have someone outside of your Penn Medicine care team support you in your everyday life?
  - Support Partner (listed and not used)

- We noticed that you listed a support partner (or partners) who we were not able to get in touch with. How do you think your program may have been different had we been able to enroll one of your support partners?
- Outside of this program, did you have other people in your life to support you with your hypertension management?
  - *If yes:* How did they help you? (ex, conversations at home, other messages etc)
  - *If no:* Would you have liked to have someone outside of your Penn Medicine care team support you in your everyday life?

- Support Partner (not listed)
  - We noticed that you did not list a support partner (or partners), is there a reason why?
  - Were you comfortable with the idea of having a support partner?
  - Outside of this program, did you have other people in your life support you with your hypertension management?
    - *If yes:* How did they help you? (ex, conversations at home, other messages etc)
    - *If no:* Would you have liked to have someone outside of your Penn Medicine care team support you in your everyday life?

Intervention: Clinical 2-way texting and visit/engagement with care team

- As a reminder, your care team was able to contact you via the study text-messaging platform to obtain additional information, provide additional guidelines for the management of your blood pressure, or set up a phone consultation. Your care team may have also requested a virtual or in-person office visit to follow-up on your medications and perform additional diagnostics to help manage your blood pressure.
  - We noticed that there wasn't very much/any response to messages and outreach from the care team. Can you tell me about this? We won't share this information directly. Your response is completely confidential.
  - Currently, text messaging from providers is not the norm, so how did you feel about receiving a text message from your care team? Although the person texting you is a provider at Penn Family Care and working in collaboration with your personal physician, they may not have been your personal physician- what are your thoughts about receiving care from a team like this, or from someone who is not normally involved in your regular care? Would you have interacted with the program more if messaging came directly from your PCP?
  - Was it difficult to keep up with the messaging you were receiving from your clinical care team?
  - Did you trust the messages? Why or why not?
  - Is there another method of communication that would have been more helpful to you?

  - During the duration of your enrollment, did you have an in person or virtual visit with either your provider or another member of your care team for management of your hypertension?
  - Did the program influence how often you scheduled visits with your PCP? (If yes) Tell me more about this.

Intervention: Medication adjustment

- Your care team may have changed the dosing of your existing medications, changed your medications, or prescribed additional medications to help manage your blood pressure. Keeping in mind that I am not a physician, I'm going to ask you some questions about having your medication adjusted:
  - Did you implement the medication change(s) suggested by your care team? If no, why not?
  - Was it an easy process? What concerns did you have, if any? And were they addressed?
  - How did you feel about having your medications changed multiple times?

Ending Questions

- In what ways did you find remote monitoring most helpful?
- What could we have done differently? What additional steps could we have taken to help you?
- Was it clear, at each point of the study, what was expected of you? If not, what did you need more help with?
- Did you notice any changes in your habits/lifestyle management due to this study? examples/why/why not?
- Would you have preferred the length of the remote monitoring program be different? In what way? Please describe.
- On a scale of 1-10, how likely are you to recommend the remote monitoring program to a friend or family member who may need this?
- Is there anything else you'd like to share with us about the program, the way you manage your blood pressure, or how we could have improved upon your program?

<u>No BP Engagement</u>
Submitted no BPs at all, no care team.

Introduction

Hello, my name is (research staff name). I am calling from the Support BP Program at Penn Medicine. Is this _____?

We are conducting a brief interview to learn more about your most recent experience with your care team at Penn Family Care in the management of your hypertension (high blood pressure). It will take approximately 20-30 minutes to complete this interview and you will receive $50 for your time to complete it. Are you willing to participate?

If yes,

Thank you for agreeing to participate! This call will be recorded to accurately keep track of the feedback you provide, but your responses will be confidential. Loss of confidentiality is possible, but unlikely. We will minimize this risk by using de-identified information and by maintaining all information on Penn

Medicine's secure drive. You may stop the interview at any time without any consequence to you. Do you agree to this phone call being recorded?

If no,

Thank you so much for your time, and have a wonderful rest of your day.

If yes,

The recording will begin now. Thank you so much for agreeing to participate in this interview.

Background

You were asked to participate in the Support BP Program because we were testing ways to help people improve their health and better manage their blood pressure. With the help of your care team at Penn Family Care, we asked you to submit blood pressure measurements over the course of 6 months to monitor and help in managing your health.

Non-Engagement Questions

- We noticed that you didn't really participate in the program by responding to the text message prompts to submit your BP measurements. What happened or changed between when you consented and when the program started?
- Please tell me about any barriers that may have prevented you from engaging with the program? (ex: issues with technology/cell phone)
- What would have made participation in the program easier for you?
- Are there other methods that you're using to monitor and manage your blood pressure?
- Is there anything that the study team could have changed in order to make sure you were engaged in the program? (ex: more detailed explanations of the intervention methods? Communication?)

Ending Questions

- What could we have done differently? What additional steps could we have taken to help you?
- Would you have preferred the length of the remote monitoring program be different? In what way? Please describe.
- On a scale of 1-10, how likely are you to recommend the remote monitoring program to a friend or family member who may need this?
- Is there anything else you'd like to share with us about your experience with the program, the way you manage your blood pressure, or how we could have improved upon your program?
