# Supplementary material for: Behavioral Economic Framing for Enrollment and Retention of Patients in Remote Blood Pressure Monitoring: A Randomized Clinical Trial
Source: JAMA Netw Open. 2025 Sep 2;8(9):e2529825. doi: 10.1001/jamanetworkopen.2025.29825 (PMC12406065; doi:10.1001/jamanetworkopen.2025.29825)
Supplement: Supplement 2. — eTable 1. Characteristics of Consented Participants in Intervention Arms eTable 2. Average Number of Blood Pressure (BP) Readings Submitted by Arm eTable 3. Rate of Engagement in BP Monitoring by Arm (at Least 50% of BPs Submitted) eTable 4. Mean Diastolic Blood Pressure and Differences by Arm eTable 5. Patient Submitted Blood Pressures by Arm eTable 6. Patient Reported Medication Adherence by Arm [file jamanetwopen-e2529825-s002.pdf]

Supplementary Online Content

Mehta SJ, Teel J, Okorie E, et al. Behavioral economic framing for enrollment and retention of patients in remote blood pressure monitoring: a randomized clinical trial. *JAMA Netw Open*. 2025;8(9):e2529825. doi:10.1001/jamanetworkopen.2025.29825

eTable 1. Characteristics of Consented Participants in Intervention Arms

eTable 2. Average Number of Blood Pressure (BP) Readings Submitted by Arm

eTable 3. Rate of Engagement in BP Monitoring by Arm (at Least 50% of BPs Submitted)

eTable 4. Mean Diastolic Blood Pressure and Differences by Arm

eTable 5. Patient Submitted Blood Pressures by Arm

eTable 6. Patient Reported Medication Adherence by Arm

This supplementary material has been provided by the authors to give readers additional information about their work.

© 2025 Mehta SJ et al. *JAMA Network Open*.

eTable 1. Characteristics of consented participants in intervention arms

	Study Arm			
	**Opt-In (n=58)**	**Opt-Out (n=63)**	**Control**	**Overall (n=121)**
Age, mean (std)	48.8 (11)	51.3 (11.2)	NA	50.1 (11.1)
Sex, n (%)				
Female	38 (65.5)	39 (61.9)	NA	77 (63.6)
Male	20 (34.5)	24 (38.1)	NA	44 (36.4)
Race, n (%)				
Asian	3 (5.2)	1 (1.6)	NA	4 (3.3)
Black	50 (86.2)	53 (84.1)	NA	103 (85.1)
Other/Unknown [a]	2 (3.4)	3 (4.8)	NA	5 (4.2)
White	3 (5.2)	6 (9.5)	NA	9 (7.4)
Ethnicity, n (%)				
Hispanic/Latino	4 (6.9)	0 (0)	NA	4 (3.3)
Not Hispanic/Latino	54 (93.1)	63 (100)	NA	117 (96.7)
Unknown	0 (0)	0 (0)	NA	0 (0)
Insurance, n (%)				
Private/Self-Pay	34 (58.6)	27 (42.9)	NA	61 (50.4)
Medicare	4 (6.9)	13 (20.6)	NA	17 (14)
Medicaid	20 (34.5)	23 (36.5)	NA	43 (35.5)
BMI, mean (std)	33.6 (6.7)	32.6 (6.4)	NA	33.1 (6.6)
Diabetes, n (%)	22 (37.9)	24 (38.1)	NA	46 (38)
Kidney Disease, n (%)	6 (10.3)	8 (12.7)	NA	14 (11.6)
Eligibility Systolic BP, mean (std)	150.9 (13.9)	150.5 (11.5)	NA	150.7 (12.7)
Eligibility Diastolic BP, mean (std)	90.8 (11.6)	90.3 (8.3)	NA	90.5 (10)

[a] "Other" includes patients who self-identified as Pacific Islander, Other, or More than 1 Race in the EHR. "Unknown" includes missing data as well as those who were self-identified as "Unknown."

© 2025 Mehta SJ et al. *JAMA Network Open*.

eTable 2. Average number of blood pressure (BP) readings submitted by arm

	**Study Arm**			
	**Opt-In (n=58)**	**Opt-Out (n=63)**	**Control (NA)**	**Overall**
Submitted BPs, Mean (SD)[a]	40.5 (28.1)	39.3 (27.2)	NA	39.9 (27.5)
Model (Arm 1 as reference)		-0.03, (-0.09, 0.03)		
p-value		0.30		
Model, Adjusted (Arm 1 as reference)[b]		-0.04 (-0.10, 0.02)		
p-value		0.16		

[a] Denominators are for consented

[b] Adjusted for age, BMI, sex, race, insurance type, and diabetes and kidney disease status

© 2025 Mehta SJ et al. *JAMA Network Open*.

eTable 3. Rate of engagement in BP monitoring by arm (at least 50% of BPs submitted)

	**Study Arm**			
	**Opt-In (n=58)**	**Opt-Out (n=63)**	**Control (NA)**	**Overall**
Engaged (submitted>50%), n (%)[a]	30 (51.7%)	33 (52.4%)	NA	63 (52.1%)
Difference, 90% CI (Arm 1 as reference)		-0.7 (-15.6-14.3)		
p-value		0.94		
Logistic Model (Arm 1 as reference)		1.03 (0.5-2.1)		
p-value		0.94		
Logistic Model, Adjusted (Arm 1 as reference)[b]		0.88 (0.4-1.96)		
p-value		0.76		

[a] Denominators are for consented

[b] Adjusted for age, BMI, sex, race, insurance type, and diabetes and kidney disease status

© 2025 Mehta SJ et al. *JAMA Network Open*.

eTable 4. Mean Diastolic Blood Pressure and Differences by Arm

**Mean Diastolic Blood Pressure**					
		**Arm**			
**Diastolic Mean BP**		**Opt-In (n=112)**	**Opt-Out (n=115)**	**Control (n=56)**	
Eligibility DBP (SD)		89.3 (11.7)	88 (14)	90.1 (11.2)	
Final DBP (SD)		83.4 (10.1)	82.7 (10.3)	85.9 (9.4)	
		**Mean Differences**			
		**Opt-In**		**Opt-Out**	
**Reference Group**	**Model**	**Mean (95% CI)**	**P-value**	**Mean (95% CI)**	**P-value**
Control	Unadjusted	-1.77 (-4.6,1.06)	0.22	-2.09 (-4.92,0.74)	0.15
	Adjusted [a]	-1.39 (-4.13,1.34)	0.32	-1.95 (-4.69,0.78)	0.16
	Imputed [b]	-1.8 (-4.4, 0.9)	0.19	-1.8 (-4.4, 0.8)	0.18
Opt-In	Unadjusted	--	--	-0.32 (-2.62,1.98)	0.78
	Adjusted [a]	--	--	-0.56 (-2.83,1.7)	0.63
	Imputed [b]	--	--	0 (-2.4, 2.3)	0.98

[a] Adjusted for age, BMI, sex, race, insurance type, and diabetes and kidney disease status
[b] Imputed with 20 imputations using predictive mean matching

© 2025 Mehta SJ et al. *JAMA Network Open*.

eTable 5. Patient submitted blood pressures by arm

	N[1]	Starting Systolic Mean (SD), mmHg	Ending Systolic Mean (SD), mmHg	Starting Diastolic Mean (SD), mmHg	Ending Diastolic Mean (SD), mmHg	Change in Systolic, mmHg	Change in Diastolic, mmHg
**Opt-In**	46	132.5 (13.6)	129.5 (14.3)	86.1 (8.7)	83.6 (7.8)	-3.0	-2.5
**Opt-Out**	51	134.2 (16.8)	127.8 (11.1)	86.7 (10.6)	82.3 (8.5)	-6.4	-4.4

[1] Only patients submitting at least 6 BPs (3 start, 3 final) included, regardless of time in program

© 2025 Mehta SJ et al. *JAMA Network Open*.

eTable 6. Patient reported medication adherence by arm[1]

	N	# Days Adherent, Mean (SD)	# Days Adherent, Median (IQR)
**Opt-in**	53	6.3 (1.4)	6.9 (6.4 – 7)
**Opt-out**	53	6.4 (1.0)	6.9 (6.2 – 7)

[1] Average patient response to the weekly prompt of "How many days out of the last 7 days did you take all your BP medication(s) as prescribed? Please input a single number 0-7"

© 2025 Mehta SJ et al. *JAMA Network Open*.
