# Supplementary material for: Behavioral Economic Framing for Enrollment and Retention of Patients in Remote Blood Pressure Monitoring: A Randomized Clinical Trial
Source: JAMA Netw Open. 2025 Sep 2;8(9):e2529825. doi: 10.1001/jamanetworkopen.2025.29825 (PMC12406065; doi:10.1001/jamanetworkopen.2025.29825)
Supplement: Supplement 3. — Data Sharing Statement [file jamanetwopen-e2529825-s003.pdf]

Data Sharing Statement

Mehta. Behavioral Economic Framing for Enrollment and Retention of Patients in Remote Blood Pressure Monitoring. *JAMA Netw Open*. Published September 02, 2025. doi:10.1001/jamanetworkopen.2025.29825

Data
Additional Information: clinicaltrials.gov NCT04714398
Data available: No

Additional Information
Explanation for why data not available: Data will not be shared as we did not receive approval from our institution.
